# Trends in Age and Socioeconomic Inequalities in Telemedicine Adoption in Japan, 2020-2024: Longitudinal Panel Study

**DOI:** 10.2196/99994

**Published:** 2026-10-08

**Authors:** Hiroshi Ito, Hirokazu Tanaka, Takahiro Tabuchi, Atsushi Miyawaki

**Affiliations:** 1 Department of Hospital Medicine University of Tsukuba Hospital Tsukuba, Ibaraki Japan; 2 Health Services Research and Development Center University of Tsukuba Tsukuba, Ibaraki Japan; 3 Division of Population Data Science National Cancer Center Institute for Cancer Control Chuo-ku, Tokyo Japan; 4 Division of Epidemiology, School of Public Health Tohoku University Graduate School of Medicine Sendai, Miyagi Japan; 5 Department of Public Health and Health Policy, Institute of Medicine University of Tsukuba Tsukuba, Ibaraki Japan

**Keywords:** telemedicine, health inequalities, socioeconomic factors, aging, longitudinal study

## Abstract

**Background:**

Theoretical perspectives offer contrasting predictions regarding how inequalities in health technology adoption evolve over time. While classical diffusion of innovations theory suggests that early adoption gaps may narrow as technologies such as telemedicine become widespread, the inverse equity hypothesis posits that such disparities are likely to persist or even widen. Longitudinal evidence on the long-term evolution of individual telemedicine adoption, however, remains scarce.

**Objective:**

This study aims to assess changes in age and socioeconomic inequalities in telemedicine adoption in Japan from 2020 to 2024.

**Methods:**

We used data from a nationwide, internet-based panel survey of the general population in Japan. Participants aged 18-75 years who had completed both the 2020 baseline and the 2024 follow-up surveys were included. The primary outcome was self-reported telemedicine adoption, harmonized as cumulative ever use at each survey wave. For each age and socioeconomic indicator, we fitted a separate inverse probability-weighted multivariable logistic regression model incorporating the exposure, survey wave, and their interaction, and adjusted for baseline demographic, socioeconomic, and health-related characteristics. Predictive margins were used to estimate adjusted prevalence and probability-scale difference-in-differences (DIDs).

**Results:**

The study included 10,818 participants (mean age 49.7, SD 16.8 years; 50.6% [unweighted n/N 5011/10,818] women). In the unweighted counts, 282 (2.6%) participants had adopted telemedicine by 2020, with this figure increasing to 758 (7%) participants by 2024. Telemedicine adoption was generally less prevalent in older age groups in 2020. Although adoption increased across all age groups, the increase was smaller among older participants (70-75 years: +1.0 percentage point [PP] vs 18-29 years: +13.1 PP; DID −12.1 PP, 95% CI −18.2 to −5.9). The increase was also smaller among participants who were unemployed at baseline than among upper nonmanual workers (+2.8 vs +5.7 PP; DID −2.9 PP, 95% CI −4.6 to −1.2). DIDs for educational attainment, urbanicity, and household income were not statistically distinguishable from zero. The findings remained largely unchanged after several sensitivity analyses.

**Conclusions:**

Between 2020 and 2024, telemedicine adoption inequalities by age and socioeconomic status showed no evidence of narrowing, and age-related inequalities widened. These findings suggest that the broader diffusion of telemedicine has not necessarily been accompanied by equitable adoption, and highlight the potential importance of targeted assistance, inclusive platform design, and support for digital access and literacy among populations facing barriers to telemedicine adoption.

## Introduction

Telemedicine has emerged as an important modality of health care delivery, offering an alternative or complement to in-person clinical encounters [1]. Its adoption accelerated during the COVID-19 pandemic, in response to the need to maintain access to health care while minimizing the infection risks associated with outpatient visits [2,3]. Telemedicine can achieve clinical effectiveness comparable to in-person care for conditions such as hypertension, dyslipidemia, diabetes, and mental health disorders [4-6], and may also alleviate access barriers related to geographic or social constraints [7]. During the early phase of the COVID-19 pandemic, telemedicine adoption expanded rapidly [8,9]. Nevertheless, cross-sectional studies conducted during this period documented inequalities in telemedicine adoption related to age and socioeconomic status (SES) [10,11], suggesting that the diffusion of telemedicine does not necessarily translate into equitable access to health care.

Research examining how these inequalities have changed over time, as telemedicine has transitioned into routine care, remains limited, however. Theoretical perspectives offer contrasting predictions regarding the longitudinal trajectory of health technology adoption. On the one hand, classical innovation diffusion frameworks suggest that, although technological advances may initially exacerbate access gaps, such disparities are likely to narrow as a technology becomes more broadly adopted and normalized [12]. On the other hand, the inverse equity hypothesis posits that early adoption disproportionately benefits socioeconomically advantaged groups, with inequalities potentially being sustained or even widening during the diffusion process [13]. Empirical evidence on how such inequalities evolve over time also remains limited. Previous longitudinal studies have largely focused on the earlier phases of the COVID-19 pandemic or on specific populations and clinical settings, rather than examining long-term changes in age and socioeconomic inequalities in telemedicine adoption among the general population [8,9,14,15]. In Japan specifically, the available evidence includes an early cross-sectional study based on a nationwide internet survey of the general population conducted in 2020 [16], and a recent claims-based study describing age-stratified trends in telemedicine use over time [17]. The former did not examine within-person changes beyond 2020, while the latter relies on aggregated claims data and does not include individual-level socioeconomic measures. Thus, it remains unclear whether age- and socioeconomic-related gaps in cumulative telemedicine adoption have narrowed or widened over the long term at the individual level.

To address these gaps, we assessed long-term changes in age- and SES-related inequalities (educational attainment, rural vs urban residence, equivalized household income, and occupational class) in telemedicine adoption, based on a nationwide longitudinal panel of the general population in Japan from 2020 to 2024. We examined whether longitudinal changes in telemedicine adoption differed across age and socioeconomic strata.

## Methods

### Study Design, Setting, and Data Sources

We conducted a longitudinal study using data from the Japan “COVID-19 and Society” Internet Survey (JACSIS). The JACSIS is a self-administered, internet-based survey, administered via an online research panel provided by Rakuten Insight, Inc, a major Japanese internet research agency that maintains a large panel of approximately 2.2 million registered individuals [18]. JACSIS has been conducted repeatedly since 2020 as a series of nationwide surveys using comparable sampling methods [19]. This study used data from the baseline survey in 2020 and the follow-up in 2024.

The baseline JACSIS survey was conducted between August 25 and September 30, 2020. Questionnaires were distributed to individuals selected from the survey panel using stratified sampling, based on sex, age group, and prefecture, with the aim of approximating the population distribution of Japan. Invitations were sent until the predetermined target number of respondents in each sex, age, and prefecture stratum was reached. Individuals who consented to participate in the survey accessed the designated website and responded to questionnaires. They also had the option not to respond or to discontinue at any point in the survey; in such cases, they were regarded as not having consented to participate in the survey and were not counted as respondents. During this period, questionnaires were distributed to 224,389 individuals, with 28,000 respondents completing the survey, corresponding to an overall response rate of 12.5% [16]. The online questionnaires covered a wide range of sociodemographic, socioeconomic, lifestyle, and health-related characteristics. There were no missing values due to the survey design described previously (if any item was not answered, the survey could not be completed). The follow-up survey was conducted between December 2, 2024, and January 16, 2025, and included participants from previous JACSIS waves, using the same consent procedure described above, resulting in no item-level missing data.

### Ethical Considerations

This study was conducted in accordance with the Declaration of Helsinki. The original JACSIS study protocol was approved by the Research Ethics Committee of the Osaka International Cancer Institute on June 19, 2020 (approval number 20084), before data collection commenced. This study was approved by the Ethics Committee of the Faculty of Medicine, University of Tsukuba on October 28, 2024 (approval number 1737-3). All the participants who completed the surveys provided electronic informed consent. Participants received compensation through Rakuten Insight’s E-point credit system, which could be used for online shopping or converted to cash. This study follows the STROBE (**Strengthening the Reporting of Observational Studies in Epidemiology**) reporting guidelines for observational studies. The completed STROBE checklist is provided in Multimedia Appendix 1.

### Study Population

To be eligible for the longitudinal study, respondents needed to have completed both the 2020 and the 2024 surveys. Among the 28,000 respondents to the 2020 survey, 2518 were excluded because of potentially unreliable or internally inconsistent responses, according to a predefined algorithm used in previous JACSIS studies (Table S1 in Multimedia Appendix 2, which provides supplementary methods, tables, and figures for the study). This left 25,482 valid baseline respondents. Of these, 10,854 respondents completed the 2024 follow-up survey, while 14,628 did not. The latter were therefore excluded from this study. After the further exclusion of 36 participants aged 15-17 years at baseline, the final analytic sample comprised 10,818 adults aged 18-75 years at baseline (Figure 1). The number of individuals specifically invited or recontacted for the 2024 follow-up and individual reasons for follow-up nonparticipation were unavailable.

**Figure 1:**
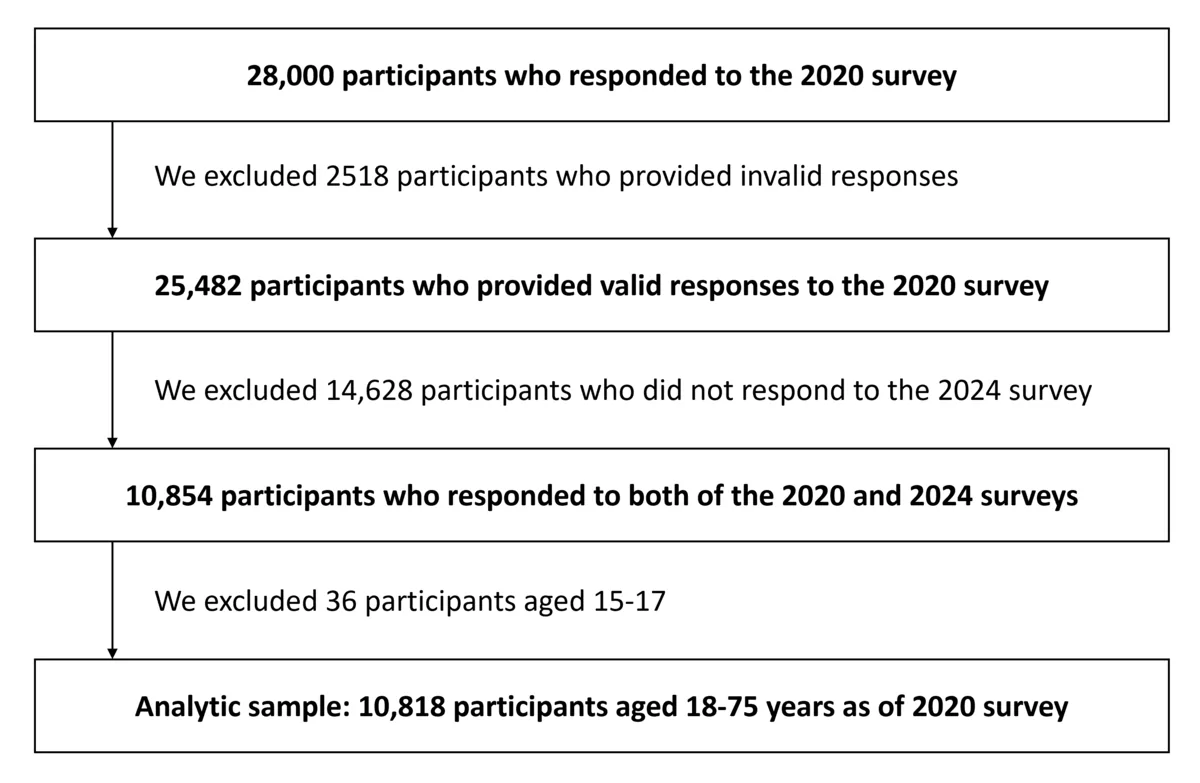
Selection of participants (numbers presented in the flowchart represent the unweighted participant counts).

### Exposure Variables

The exposure variables were baseline age and indicators of SES. Age at baseline was categorized into 6 groups (18-29 [reference category], 30-39, 40-49, 50-59, 60-69, and 70-75 years). Indicators of SES, all measured in the 2020 baseline survey, included educational attainment, rural versus urban residence, equivalized household income, and occupational class [20]. Educational attainment was categorized into 3 groups, according to the International Standard Classification of Education: university degree or higher (reference category), 2-year college degree, and high school diploma or lower [21]. Residence was dichotomized as urban (reference category) or rural, based on the respondent’s residential area, as determined from their reported 7-digit zip code. Areas defined as densely inhabited districts in the official documentation for the 2015 Population Census of Japan were classified as urban; all other areas were classified as rural [22]. Equivalized household income was calculated by dividing self-reported annual household income by the square root of the household size. It was categorized, using prespecified cutoffs, as high (≥4.30 million Japanese yen [JPY] [reference category]), medium (≥2.50 to <4.30 million JPY), low (<2.50 million JPY), or not answered. The “not answered” category comprised participants who selected either “prefer not to answer” or “do not know.” For reference, the average exchange rate in 2020 was approximately JPY 106.8 per US $1. Occupational class was categorized as upper nonmanual workers (reference category), lower nonmanual workers, manual workers, self-employed (including farmers), and unemployed [23]. Occupational status was defined at baseline; subsequent transitions in employment were not captured.

Each exposure variable was examined separately. When evaluating a given exposure (eg, educational attainment), age and the other SES measures were included as adjustment variables. The models were intended to describe adjusted inequalities and temporal changes, rather than to estimate the causal effects of age or SES.

### Outcome Variables

The primary outcome was cumulative telemedicine adoption, defined as ever having used telemedicine by the time of each survey wave. Rather than looking at cross-sectional use at each time point, we examined cumulative adoption in order to capture the diffusion of telemedicine over time. In 2020, participants were asked whether they had experienced telemedicine since April 2020, and given a choice of 1 out of 3 responses: first-time use, previous use, or no use. The first-use and previous-use responses were classified as ever use by 2020.

In 2024, the participants were asked to select 1 option from 5 responses: first use within the most recent 2 months; first use within the past year; use within the past year but not for the first time; use more than 1 year earlier but not within the past year; or no previous use. The first 4 responses were classified as ever use by 2024. To preserve the cumulative interpretation of ever use, participants classified as ever users in 2020 were also classified as ever users in 2024, even when their 2024 response was inconsistent with their earlier report; this was termed the carry-forward rule (Table S2 in Multimedia Appendix 2).

### Adjustment Variables

The primary estimands were differences in temporal changes in telemedicine adoption associated with age or SES, after adjusting for potential confounders. We adjusted for the participants’ demographic and health-related characteristics, as derived from the 2020 survey. Demographic characteristics included sex, marital status (married, never married, widowed, and separated), and household size (1, 2, 3, 4, or ≥5 household members). Health-related characteristics included smoking status (never, ever, and current), walking disability (defined as any reported difficulty in walking), and self-reported physician-diagnosed comorbidities, including hypertension, diabetes, psychological disorder, asthma, coronary artery disease, cancer, stroke, and chronic obstructive pulmonary disease.

### Statistical Analysis

First, the demographic, socioeconomic, and health-related characteristics of the study participants as of the 2020 survey were reported. Unless otherwise specified, the analyses were weighted by the analytic weight to minimize sampling bias and selection bias. The analytic weight was constructed as the product of the baseline sampling weight and an inverse probability-of-analytic-inclusion weight. The baseline sampling weight was intended to account for differences between participants in the 2020 internet survey and the general population. The inverse propensity scores for baseline participation were estimated using demographic, socioeconomic, lifestyle, and health-related characteristics by comparing JACSIS respondents with participants in the nationally representative 2016 Comprehensive Survey of Living Conditions [24,25]. The inverse probability-of-analytic-inclusion weight was calculated by first estimating the probability of inclusion in the longitudinal analytic sample for all the 28,000 baseline respondents. Inclusion was coded as 1 for the 10,818 participants included in the longitudinal analysis. The model included all baseline adjustment variables, including baseline telemedicine adoption [26]. The baseline sampling weight and the inverse probability-of-analytic-inclusion weight were multiplied, and the resulting final weight was normalized to have a mean of 1.000 (SD 1.417). Detailed information on weight construction is provided in Methods S1 in Multimedia Appendix 2. Distributions of the component and final weights and diagnostics for the predicted analytic-inclusion probabilities, including ranges, selected percentiles, coefficients of variation, effective sample sizes, and threshold counts, are reported in Table S3 in Multimedia Appendix 2.

Second, we tabulated the number of events and weighted prevalence for each exposure group in each survey wave to assess whether there were sufficient observations within each group and wave.

Third, we examined changes in telemedicine adoption between 2020 and 2024 according to age. We first estimated the unadjusted changes using unweighted and weighted logistic regression models, including the exposure of interest (age group), the survey wave, and their interaction terms. We then fitted a fully adjusted weighted multivariable logistic regression model using the same specification, adjusting additionally for the remaining exposure (SES measures) and the adjustment variables described above. Marginal standardization was used to estimate the adjusted prevalence of telemedicine adoption in each age group and survey wave. Within-age-group change was calculated as the difference in the adjusted prevalence between 2024 and 2020. Differences-in-differences (DIDs) were then calculated as the differences in temporal change between each age group and the prespecified reference group (18-29 years). CIs and *P* values for within-group changes and DIDs were calculated directly from the linear contrasts of the predictive margins using the delta method, rather than from the coefficients of the logit-scale interaction terms. SEs in the primary analysis were clustered at the prefecture level (47 clusters) to account for correlations among observations from participants residing in the same prefecture.

Fourth, to examine changes in telemedicine adoption according to SES, we fitted separate weighted multivariable logistic regression models for each SES measure (educational attainment, urbanicity, equivalized household income, and occupational class). These included the SES measure of interest, the survey wave, their interaction terms, and the remaining exposure and adjustment variables described above. Adjusted prevalences, within-group changes, and DIDs were estimated using the procedures described above.

All analyses were conducted using Stata (version 18.0; StataCorp LLC). All the tests were 2-sided. To account for multiple comparisons across the 15 nonreference contrasts (5 age, 2 educational attainment, 1 urbanicity, 3 household income, and 4 occupational class contrasts), Benjamini-Hochberg (BH) false discovery rate–adjusted *P* values were calculated across the 15 contrasts. These are reported together with the unadjusted *P* values. Statistical significance was defined as BH-adjusted *P* values of <.05. To assess the statistical power to detect differences in temporal changes, for each of the 15 nonreference DIDs, an approximate minimum detectable effect for 80% power at a 2-sided α of .05 was calculated as (1.96 + 0.84) multiplied by the SE of the DID.

### Sensitivity Analyses

We conducted a number of sensitivity analyses. First, we repeated the analyses using alternative model specifications, as follows: no weighting (unweighted fully adjusted model); a narrower set of adjustment variables (because health-related and household variables might lie on pathways linking SES to telemedicine adoption), including only sex in age-related models, and sex and age in SES-related models (minimally adjusted weighted models); a linear regression model instead of a logistic regression model (weighted linear probability model); participant-level clustering instead of prefecture-level clustering; and winsorization of the final weight at the 1st and 99th percentiles. Second, instead of treating item nonresponse for income (ie, participants who selected either “prefer not to answer” or “do not know”) as a separate “not answered” category, we conducted a complete-case analysis (restricting the sample to respondents with valid income data) modeling income as tertiles, a complete-case analysis modeling income as a continuous variable, and a multiple imputation of income nonresponse. Third, we restricted the sample to participants who were nonusers in the 2020 survey, and examined new adoption by 2024. Fourth, we adjusted additionally for digital access (proxied by baseline ownership of a smartphone, tablet, and personal computer, and home internet access). Since this could be either a confounder or a mediator, it was not included in the primary analysis. Fifth, we used an alternative outcome definition that did not carry forward telemedicine use from 2020 to 2024; specifically, participants who had ever used telemedicine in 2020 but reported no use in 2024 were classified as nonusers in 2024. The details of these analyses are described in Methods S1 in Multimedia Appendix 2.

### Secondary Analyses

Because the unemployed category encompassed heterogeneous social groups, a supplementary analysis was conducted separating this category into students, retired participants, homemakers, and other participants not employed. We also examined past-year telemedicine use in 2024 among participants who were nonusers in 2020. This outcome reflects recent telemedicine use rather than cumulative adoption, and was therefore considered complementary to the primary ever-use outcome.

## Results

### Characteristics of the Participants

The analytic sample consisted of 10,818 participants who had completed both the 2020 baseline survey and the 2024 follow-up (Figure 1). The mean age of the participants was 49.7 (SD 16.8) years, and 50.6% (unweighted n/N=5011/10,818) of them were women (Table 1). More than half of the participants had an educational attainment of high school diploma or lower, 38.9% (unweighted n/N=3276/10,818) resided in rural areas, and 39% (unweighted n/N=4132/10,818) were unemployed as of the 2020 survey. In the unweighted counts, 282 (2.6%) participants had adopted telemedicine by 2020, with this figure increasing to 758 (7%) participants by 2024, and there were no exposure groups or survey waves with sparse outcome events (Table S4 in Multimedia Appendix 2).

**Table 1:** Characteristics of the participants in 2020.

Characteristics			Weighted value (N=10,818)			
Female, n (%)			5477 (50.6)			
Age (years), mean (SD)			49.7 (16.8)			
**Educational attainment, n (%)**						
	University degree or higher			2817 (26.0)		
	2-year college degree			2203 (20.4)		
	High school diploma or lower			5797 (53.6)		
**Urbanicity of residence, n (%)**						
	Urban			6614 (61.1)		
	Rural			4204 (38.9)		
**Household equivalized income (JPY^a,b^), n (%)**						
	High (≥4.30 million)			2239 (20.7)		
	Medium (2.50-4.30 million)			2769 (25.6)		
	Low (<2.50 million)			3422 (31.6)		
	Not answered			2388 (22.1)		
**Occupational class, n (%)**						
	Upper nonmanual workers			2374 (21.9)		
	Lower nonmanual workers			1902 (17.6)		
	Manual workers			1580 (14.6)		
	Self-employed (including farmers)			745 (6.9)		
	Unemployed			4217 (39.0)		
**Marital status, n (%)**						
	Married			7096 (65.6)		
	Never married			2523 (23.3)		
	Widowed			580 (5.4)		
	Separated			619 (5.7)		
**Household size, n (%)**						
	1			1675 (15.5)		
	2			3784 (35.0)		
	3			2492 (23.0)		
	4			1939 (17.9)		
	≥5			928 (8.6)		
**Smoking status, n (%)**						
	Never			5676 (52.5)		
	Ever			2955 (27.3)		
	Current			2187 (20.2)		
Walking disability, yes, n (%)			1109 (10.2)			
**Comorbidities, n (%)**						
	Hypertension			2216 (20.5)		
	Diabetes			908 (8.4)		
	Psychological disorder			634 (5.9)		
	Asthma	490 (4.5)				
	Coronary disease	350 (3.2)				
	Cancer	303 (2.8)				
	Stroke	239 (2.2)				
	COPD^c^	176 (1.6)				

^a^JPY: Japanese yen.

^b^The average exchange rate in 2020 was approximately JPY 106.8 per US $1.

^c^COPD: chronic obstructive pulmonary disease.

Inverse probability weighting was applied to account for baseline selection into the internet survey and inclusion in the final longitudinal analytic sample, including follow-up nonparticipation and prespecified analytic exclusions. Participants were considered to have walking disability if they reported any difficulty in walking around, defined as selecting any response other than “no difficulty walking around” (ie, slight, moderate, severe difficulty, or inability to walk).

### Age-Related Differences in Telemedicine Adoption

Results from the unadjusted models are presented in Table S5 in Multimedia Appendix 2. In 2020, after adjustment for potential confounders, the adjusted prevalences of telemedicine adoption were generally lower among older age groups than among younger groups, although the prevalence was slightly higher among participants aged 70-75 years than among those aged 60-69 years (Figure 2). Between 2020 and 2024, telemedicine adoption increased across all age groups, but smaller absolute increases were observed among the older participants. Participants aged 18-29 years showed an absolute increase of 13.1 percentage points (PP; 95% CI 7.0-19.1), whereas participants aged 70-75 years showed an increase of 1.0 PP (95% CI 0.1-1.9), yielding a DID of −12.1 PP (95% CI −18.2 to −5.9; *P*<.001; BH-adjusted *P*=.002; Table 2).

**Figure 2:**
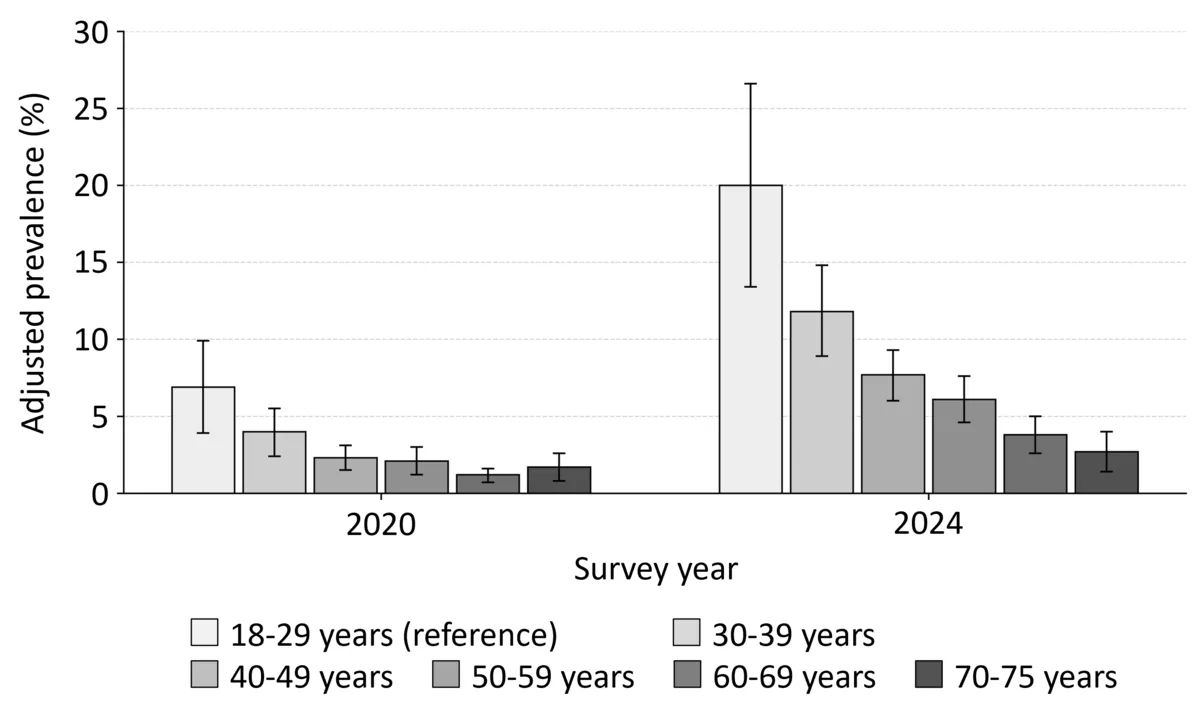
Adjusted rates of telemedicine use between 2020 and 2024 by age group (error bars indicate 95% CIs of the adjusted prevalence).

**Table 2:** Difference in the adjusted rates of telemedicine use between 2020 and 2024 by age group (difference-in-difference analysis).

Age group (y)	Weighted number of participants	Adjusted prevalence (95% CI), %			Adjusted difference (95% CI), percentage points		Difference-in-differences (95% CI), percentage points		Unadjusted *P* value		BH^a^-adjusted *P* value
		2020	2024								
18-29	1703	6.9 (3.9 to 9.9)	20.0 (13.4 to 26.6)	+13.1 (+7.0 to +19.1)		Reference^b^		—^c^		—	
30-39	1592	4.0 (2.4 to 5.5)	11.8 (8.9 to 14.8)	+7.9 (+5.5 to +10.2)		–5.2 (–11.8 to +1.4)		.12		.24	
40-49	2100	2.3 (1.5 to 3.1)	7.7 (6.0 to 9.3)	+5.4 (+4.0 to +6.9)		–7.7 (–14.2 to –1.1)		.02		.07	
50-59	1916	2.1 (1.2 to 3.0)	6.1 (4.6 to 7.6)	+4.0 (+2.9 to +5.1)		–9.1 (–15.3 to –2.9)		.004		.02	
60-69	1700	1.2 (0.7 to 1.6)	3.8 (2.6 to 5.0)	+2.6 (+1.4 to +3.7)		–10.5 (–16.7 to –4.3)		<.001		.004	
70-75	1807	1.7 (0.8 to 2.6)	2.7 (1.4 to 4.0)	+1.0 (+0.1 to +1.9)		–12.1 (–18.2 to –5.9)		<.001		.002	

^a^BH: Benjamini-Hochberg.

^b^The prespecified reference category for the difference-in-differences analysis.

^c^Not applicable.

We calculated the differences in the adjusted rates of telemedicine use between 2020 and 2024 for each age group. Then, we examined how the difference in the rates of telemedicine use between the two time points varied by age group (DIDs). Inverse probability weighting was applied to account for baseline selection into the internet survey and inclusion in the final longitudinal analytic sample, including follow-up nonparticipation and prespecified analytic exclusions. For each analysis, SEs were clustered at the prefecture level (47 clusters). Differences were calculated from the unrounded estimates; consequently, the displayed values may differ by 0.1 PP from differences calculated using the rounded prevalence estimates. BH-adjusted *P* values were calculated using the BH procedure across the 15 nonreference contrasts.

### Socioeconomic Differences in Telemedicine Adoption

In 2020, the adjusted prevalences for telemedicine adoption were lower among participants with a 2-year college degree or a high school diploma or lower than among those with a university degree or higher (Figure 3). Between 2020 and 2024, prevalence increased across all educational groups, but neither educational DID was statistically distinguishable from zero (Table 3).

**Figure 3:**
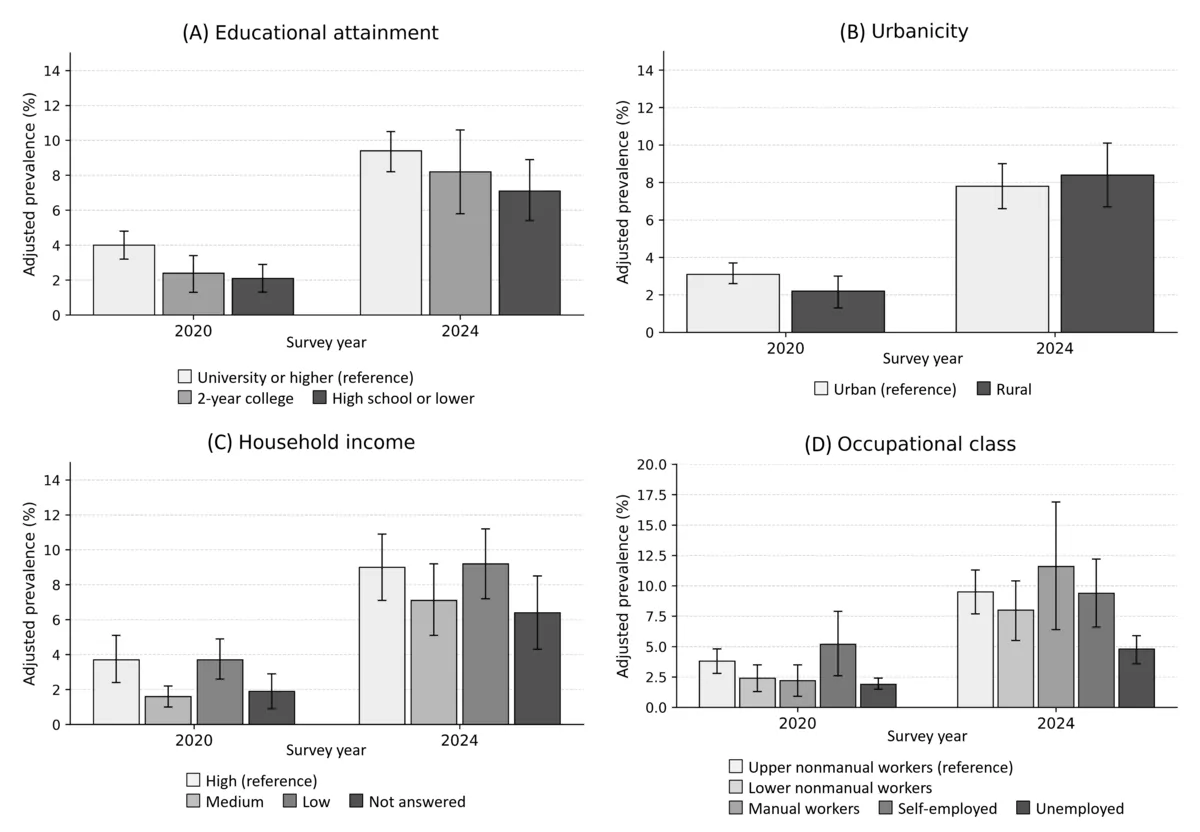
Adjusted rates of telemedicine use between 2020 and 2024 by socioeconomic status (error bars indicate 95% CIs of the adjusted prevalence).

**Table 3:** Differences in the adjusted rates of telemedicine use between 2020 and 2024 by socioeconomic status (difference-in-difference analysis).

Socioeconomic status		Weighted number of participants	Adjusted prevalence (95% CI), %		Adjusted difference (95% CI), percentage points	Difference-in-differences (95% CI), percentage points	Unadjusted *P* value	BH^a^-adjusted *P* value					
			2020	2024									
**Educational attainment**													
	University degree or higher	2817	4.0 (3.2 to 4.8)	9.4 (8.2 to 10.5)	+5.4 (+4.6 to +6.1)	Reference^b^	—^c^	—					
	2-year college degree	2203	2.4 (1.3 to 3.4)	8.2 (5.8 to 10.6)	+5.8 (+3.4 to +8.3)	+0.5 (–2.1 to +3.1)	.72	.86					
	High school diploma or lower	5797	2.1 (1.3 to 2.9)	7.1 (5.4 to 8.9)	+5.0 (+3.4 to +6.6)	–0.4 (–2.1 to +1.4)	.70	.86					
**Urbanicity of residence**													
	Urban	6614	3.1 (2.6 to 3.7)	7.8 (6.6 to 9.0)	+4.7 (+3.8 to +5.5)	Reference	—	—					
	Rural	4204	2.2 (1.3 to 3.0)	8.4 (6.7 to 10.1)	+6.2 (+4.6 to +7.9)	+1.6 (–0.1 to +3.3)	.07	.17					
**Household** **equivalized** **income**													
	High	2239	3.7 (2.4 to 5.1)	9.0 (7.1 to 10.9)	+5.3 (+4.1 to +6.4)	Reference	—	—					
	Medium	2769	1.6 (1.0 to 2.2)	7.1 (5.1 to 9.2)	+5.5 (+3.5 to +7.6)	+0.3 (–1.9 to +2.5)	.79	.86					
	Low	3422	3.7 (2.6 to 4.9)	9.2 (7.2 to 11.2)	+5.5 (+4.0 to +6.9)	+0.2 (–1.5 to +1.9)	.81	.86					
	Not answered	2388	1.9 (0.9 to 2.9)	6.4 (4.3 to 8.5)	+4.5 (+2.6 to +6.4)	-0.7 (–3.1 to +1.6)	.54	.81					
**Occupational class**													
	Upper nonmanual workers	2374	3.8 (2.8 to 4.8)	9.5 (7.7 to 11.3)	+5.7 (+4.5 to +7.0)	Reference	—	—					
	Lower nonmanual workers	1902	2.4 (1.3 to 3.5)	8.0 (5.5 to 10.4)	+5.6 (+3.5 to +7.6)	–0.2 (–2.6 to +2.2)	.88	.88					
	Manual workers	1580	2.2 (0.9 to 3.5)	11.6 (6.4 to 16.9)	+9.5 (+4.8 to +14.1)	+3.7 (–1.2 to +8.6)	.14	.24					
	Self-employed	745	5.2 (2.6 to 7.9)	9.4 (6.6 to 12.2)	+4.2 (+2.4 to +5.9)	–1.6 (–3.6 to +0.5)	.13	.24					
	Unemployed	4217	1.9 (1.5 to 2.4)	4.8 (3.6 to 5.9)	+2.8 (+1.9 to +3.8)	–2.9 (–4.6 to –1.2)	<.001	.004					

^a^BH: Benjamini-Hochberg.

^b^The prespecified reference category for the difference-in-differences analysis.

^c^Not applicable.

The adjusted prevalences were similar between urban and rural residents in both survey waves. Telemedicine adoption increased in both groups, with a rural-versus-urban DID of 1.6 PP (95% CI −0.1 to 3.3; BH-adjusted *P*=.17). For income, the adjusted prevalence increased in every category, but none of the income DIDs were statistically distinguishable from zero (Table 3).

Telemedicine adoption also increased across all occupational classes. Manual workers showed the largest absolute increase (9.5 PP, 95% CI 4.8-14.1), although the DID relative to upper nonmanual workers was not statistically significant (3.7 PP, 95% CI −1.2 to 8.6; BH-adjusted *P*=.24). The increase was significantly smaller among unemployed participants than among upper nonmanual workers (2.8 vs 5.7 PP; DID −2.9 PP, 95% CI −4.6 to −1.2; BH-adjusted *P*=.004; Table 3).

The approximate minimum detectable effects for the nonsignificant education, urbanicity, and household-income contrasts ranged from 2.4 to 3.7 PP (Table S6 in Multimedia Appendix 2), whereas the absolute values of the corresponding DID point estimates were smaller than the estimated minimum detectable effects.

We calculated the differences in the adjusted rates of telemedicine use between 2020 and 2024 for each SES measure (educational attainment, urbanicity of residence, income level, and occupation). Then, we examined how the difference in the rates of telemedicine use between the two time points varied by educational attainment, urbanicity of residence, income level, or occupation (DIDs). Inverse probability weighting was applied to account for baseline selection into the internet survey, and inclusion in the final longitudinal analytic sample, including follow-up nonparticipation and prespecified analytic exclusions, was also applied. For each analysis, SEs were clustered at the prefecture level (47 clusters). Differences were calculated from the unrounded estimates; consequently, the displayed values may differ by 0.1 PP from differences calculated using the rounded prevalence estimates. BH-adjusted *P* values were calculated using the BH procedure across the 15 nonreference contrasts in Tables 2 and 3.

### Sensitivity Analyses

Our findings were qualitatively unaffected by: the use of a number of alternative model specifications; handling missing income in alternative ways; restricting the sample to the 2020 nonusers to examine new adoption; additionally adjusting for digital access; or applying the no-carry-forward outcome definition. The direction of the age-related differences remained consistent across all of these sensitivity analyses (Tables S7-S9 and Figures S1 and S2 in Multimedia Appendix 2). The unemployed-versus-upper-nonmanual contrast was also in the same direction as in the primary analysis, although statistical significance was not maintained in the no-carry-forward analysis (Table S7 in Multimedia Appendix 2).

### Secondary Analyses

After refining the unemployed category, smaller increases in telemedicine adoption relative to upper nonmanual workers were observed among retired participants, homemakers, and other participants not employed, whereas the contrast for students was not statistically distinguishable from zero (Table S10 in Multimedia Appendix 2). In the secondary analysis of past-year telemedicine use among participants who were nonusers in 2020, participants aged 70-75 years had a lower adjusted probability of past-year use than those aged 18-29 years (risk difference [RD] −6.3 PP, 95% CI −10.5 to −2.1; BH-adjusted *P*=.030), whereas the unemployed-versus-upper-nonmanual contrast was attenuated (RD −1.3 PP, 95% CI −2.6 to 0.0; BH-adjusted *P*=.13; Table S11 in Multimedia Appendix 2).

## Discussion

### Principal Findings

Using a nationwide, internet-based survey of the general population in Japan, we found that the prevalence of telemedicine adoption increased between 2020 and 2024. However, the longitudinal changes differed noticeably by age, with older individuals experiencing smaller increases in adoption. Age-related gaps therefore widened over time. In contrast, evidence regarding socioeconomic indicators was more subtle and heterogeneous. While the primary analysis indicated a smaller increase among participants who were unemployed at baseline compared with upper nonmanual workers, changes across levels of educational attainment, urban versus rural residence, and household income were not statistically distinguishable from zero. These findings are partly consistent with the inverse equity hypothesis with respect to age, suggesting that broad technological diffusion may not necessarily be accompanied by equitable population-level uptake. The earlier, cross-sectional Japanese study [16] analyzed data from the same 2020 nationwide internet survey wave that serves as the baseline for the present longitudinal cohort. This study extends that work by following the same individuals through to 2024, and examining longitudinal changes in age- and socioeconomic-related inequalities in telemedicine adoption. Compared with the recent claims-based study [17], which described age-stratified trends without individual-level socioeconomic measures, our study provides complementary individual-level longitudinal evidence.

There are several mechanisms that may explain the smaller increase in telemedicine adoption observed among older individuals. First, older individuals have had fewer opportunities to engage with digital technologies, which may create challenges in using telemedicine platforms [27,28]. Second, age-related sensory, cognitive, and physical impairments may make it more difficult for these individuals to engage with digital health technologies [29]. Third, older individuals may be more accustomed to in-person physician consultations and have lower levels of trust in digital health tools [28,30]. Together, these age-related factors may limit the uptake of telemedicine among older individuals and may have contributed to the relatively smaller increase in telemedicine adoption observed in this age group. Similar age-related inequalities have been observed in countries other than Japan [31-33]. The age-related difference remained evident and was essentially unchanged after additional adjustments for baseline device ownership and home internet access, suggesting that these measured aspects of digital access did not materially affect the observed age gradient.

We also found that the DIDs for educational attainment, household income, and urbanicity were not statistically distinguishable from zero. Given the precision of these estimates, these findings do not exclude potentially meaningful differences in temporal change, but our data do not provide any clear evidence of differential change across these socioeconomic indicators. Regarding employment, the primary analysis indicated a smaller increase among unemployed individuals compared with upper nonmanual workers. However, while this contrast remained similar to the primary estimate after additional adjustment for digital-access proxies, it was attenuated under some alternative outcome classifications, indicating that the employment-related finding was less robust than the age-related finding. Manual workers showed a larger point increase than upper nonmanual workers, but the CI was wide and included the null value; therefore, this pattern should not be interpreted as clear evidence of narrowing occupational inequality.

Our findings have several practical implications. Interventions aimed at supporting adoption among older individuals may be important, particularly those addressing digital access and literacy [34]. Structured onboarding or telehealth navigator programs may facilitate telemedicine use [35], while community-based navigator approaches may help address digital access barriers [36].

This study has several limitations. First, residual confounding cannot be ruled out. Although baseline ownership of a smartphone, tablet, and personal computer and home internet access were available and were included in a sensitivity analysis, individual digital literacy, such as specific IT skills or frequency of internet use, was not directly measured. Thus, the available measures captured digital access rather than validated digital literacy or specific digital skills. This limited our ability to distinguish the extent to which observed inequalities reflected digital access, digital skills, or other age-related factors.

Second, the outcome measurements relied on self-reported questionnaires, and the response structure and recall periods differed between the 2020 and 2024 surveys. Although responses were harmonized as cumulative adoption status (ever-use), additional analyses using alternative outcome classifications and a secondary analysis of past-year use showed that the age-related finding remained directionally consistent, whereas the unemployment-related finding was less robust. Third, although inverse probability weighting was used, longitudinal attrition over 4 years may introduce subtle bias. Fourth, excluding individuals older than 75 years limits generalizability to the oldest-old, among whom telemedicine adoption may be influenced by caregiver or family support [17]. Because caregiver-mediated use may partly compensate for barriers relating to an older individual’s own digital skills or ability to navigate telemedicine platforms, the age gradient observed among adults aged 18-75 years may not extrapolate directly to those older than 75 years. Fifth, our outcome combined all telemedicine modalities (telephone, video, and asynchronous). However, because video visits require greater digital literacy than telephone visits, equity patterns may vary across specific modalities. Future longitudinal studies should therefore examine whether age- and socioeconomic-related inequalities differ according to telemedicine modality. Finally, internet survey sampling underrepresents individuals who completely lack any internet access, and may therefore not fully reflect the true magnitude of population-level digital disparities.

### Conclusion

Between 2020 and 2024, age-related gaps in telemedicine adoption widened, whereas DIDs by educational attainment, income, and urbanicity were not statistically distinguishable from zero. These results suggest that broader diffusion of telemedicine has not necessarily been accompanied by equitable adoption, and underscore the potential importance of targeted assistance, simple platform designs, and digital literacy support as telemedicine becomes normalized.

## Data Availability

The data supporting the findings of this study are available from the corresponding author upon reasonable request. Access to data may be subject to restrictions due to data use agreements and ethical considerations related to the Japan “COVID-19 and Society” Internet Survey (JACSIS) study.

## Appendix

Multimedia Appendix 1 STROBE checklist.

Multimedia Appendix 2 Additional details on participant exclusions, outcome harmonization, weighting diagnostics, unadjusted and sensitivity analyses, and secondary analyses.

## Abbreviations

BH: Benjamini-Hochberg
DID: difference-in-differences
JACSIS: Japan “COVID-19 and Society” Internet Survey
JPY: Japanese yen
PP: percentage point
RD: risk difference
SES: socioeconomic status
STROBE: Strengthening the Reporting of Observational Studies in Epidemiology
