# Supplementary material for: Trends in Age and Socioeconomic Inequalities in Telemedicine Adoption in Japan, 2020-2024: Longitudinal Panel Study
Source: J Med Internet Res. 2026 Oct 8;28:e99994. doi: 10.2196/99994 (PMC13691474; doi:10.2196/99994)
Supplement: Supplementary file 1 [file jmir_v28i1e99994_app1.pdf]

STROBE Statement—checklist of items that should be included in reports of observational studies

| | Item No. | Recommendation | Page No. | Relevant text from manuscript |
|---|---|---|---|---|
| **Title and abstract** | 1 | (*a*) Indicate the study's design with a commonly used term in the title or the abstract | 1 | The title identifies the study as "A Longitudinal Panel Study." |
| | | (*b*) Provide in the abstract an informative and balanced summary of what was done and what was found | 2–3 | The Abstract summarizes the background, objective, longitudinal panel design, participants, outcome, statistical methods, main results, and conclusions. |
| **Introduction** | | | | |
| Background/rationale | 2 | Explain the scientific background and rationale for the investigation being reported | 4–5 | The Introduction describes the rapid expansion of telemedicine, previously reported age and socioeconomic inequalities, competing theoretical perspectives, and the lack of long-term longitudinal evidence. |
| Objectives | 3 | State specific objectives, including any prespecified hypotheses | 5 | "We assessed long-term changes in age- and SES-related inequalities … in telemedicine adoption … from 2020 to 2024. We examined whether longitudinal changes in telemedicine adoption differed across age and socioeconomic strata." |
| **Methods** | | | | |
| Study design | 4 | Present key elements of study design early in the paper | 6 | "We conducted a longitudinal study using data from the Japan 'COVID-19 and Society' Internet Survey (JACSIS)." |
| Setting | 5 | Describe the setting, locations, and relevant dates, including periods of recruitment, exposure, follow-up, and data collection | 6 | Nationwide internet-based survey in Japan. Baseline: August 25–September 30, 2020; follow-up: December 2, 2024–January 16, 2025. |
| Participants | 6 | (*a*) *Cohort study*—Give the eligibility criteria, and the sources and methods of selection of participants. Describe methods of follow-up<br>*Case-control study*—Give the eligibility criteria, and the sources and methods of case ascertainment and control selection. Give the rationale for the choice of cases and controls<br>*Cross-sectional study*—Give the eligibility criteria, and the | 6–7 | Participants were selected from the JACSIS internet panel. Eligibility required completion of both the 2020 and 2024 surveys. Participant selection, exclusions, follow-up completion, and final analytic sample are described. |

| | | | | |
|---|---|---|---|---|
| | | sources and methods of selection of participants | | |
| | | (*b*) *Cohort study*—For matched studies, give matching criteria and number of exposed and unexposed<br>*Case-control study*—For matched studies, give matching criteria and the number of controls per case | N/A | Not applicable; this was not a matched cohort study. |
| Variables | 7 | Clearly define all outcomes, exposures, predictors, potential confounders, and effect modifiers. Give diagnostic criteria, if applicable | 7–10 | Exposure variables were baseline age and socioeconomic indicators; the outcome was cumulative telemedicine adoption; demographic and health-related adjustment variables are defined in the Methods. |
| Data sources/ measurement | 8* | For each variable of interest, give sources of data and details of methods of assessment (measurement). Describe comparability of assessment methods if there is more than one group | 8–10 | Educational attainment, urbanicity, household income, occupational class, telemedicine adoption, and adjustment variables were obtained from the self-administered JACSIS questionnaires; definitions and categorizations are described. |
| Bias | 9 | Describe any efforts to address potential sources of bias | 10–13 | Inverse probability weighting was used to reduce sampling and longitudinal selection bias. Weight diagnostics and multiple sensitivity analyses were conducted. |
| Study size | 10 | Explain how the study size was arrived at | 7, 12–13 | The final analytic sample comprised 10,818 participants meeting the longitudinal eligibility criteria. Precision was additionally assessed using minimum detectable effects for the 15 nonreference DIDs. |
| | | | | |
| Quantitative variables | 11 | Explain how quantitative variables were handled in the analyses. If applicable, describe which groupings were chosen and why | 7–8 | Age was categorized into six prespecified groups; equivalized household income was categorized using prespecified cutoffs. |
| Statistical methods | 12 | (*a*) Describe all statistical methods, including those used to control for confounding | 10–13 | Weighted multivariable logistic regression, predictive margins, probability-scale DIDs, delta-method confidence intervals and P values, prefecture-level clustering, and BH multiplicity adjustment are described. |
| | | (*b*) Describe any methods used to examine subgroups and interactions | 11–12 | Models included exposure, survey wave, and exposure-by-wave interaction terms. Separate models were fitted for age and each socioeconomic indicator. |
| | | (*c*) Explain how missing data were addressed | 8, 13 | The web-based survey required a response before proceeding, so there were no item-level missing values. “Prefer not to answer” and “do not know” for income were combined as “not answered”; additional income-nonresponse sensitivity analyses were performed. |

| | | | | |
|---|---|---|---|---|
| | | (*d*) *Cohort study*—If applicable, explain how loss to follow-up was addressed<br>*Case-control study*—If applicable, explain how matching of cases and controls was addressed<br>*Cross-sectional study*—If applicable, describe analytical methods taking account of sampling strategy | 7, 10–11 | Follow-up participation is reported. An inverse probability-of-analytic-inclusion weight was estimated among all 28,000 baseline respondents to account for inclusion in the longitudinal analytic sample. |
| | | (*e*) Describe any sensitivity analyses | 13–14 | Sensitivity analyses included alternative weighting and adjustment specifications, linear probability models, participant-level clustering, winsorized weights, alternative income analyses, digital-access adjustment, new-adoption analysis, and a no-carry-forward outcome definition. |
| **Results** | | | | |
| Participants | 13* | (a) Report numbers of individuals at each stage of study—eg numbers potentially eligible, examined for eligibility, confirmed eligible, included in the study, completing follow-up, and analysed | 7 | Numbers are reported for baseline respondents, exclusions, valid baseline respondents, follow-up completers/noncompleters, age exclusions, and the final analytic sample. |
| | | (b) Give reasons for non-participation at each stage | 7 | Reasons for prespecified exclusions are reported; the manuscript also states that individual reasons for follow-up nonparticipation were unavailable. |
| | | (c) Consider use of a flow diagram | 7; Figure 1 | Participant selection is presented in Figure 1. |
| Descriptive data | 14* | (a) Give characteristics of study participants (eg demographic, clinical, social) and information on exposures and potential confounders | 14–16; Table 1 | Participant demographic, socioeconomic, lifestyle, and health characteristics are summarized in Table 1. |
| | | (b) Indicate number of participants with missing data for each variable of interest | 8, 15–16 | There were no item-level missing values for the variables included in the analyses. Household-income nonresponse is reported separately as "Not answered." |
| | | (c) *Cohort study*—Summarise follow-up time (eg, average and total amount) | 6–7 | Participants were followed from the 2020 baseline survey to the 2024 follow-up survey; the dates of both survey waves are reported. |
| Outcome data | 15* | *Cohort study*—Report numbers of outcome events or summary measures over time | 14; eTable 4 | "282 participants (2.6%) had adopted telemedicine by 2020," increasing to "758 participants (7.0%) by 2024." Group- and wave-specific events and weighted prevalence are reported in eTable 4. |

| | | | | |
|---|---|---|---|---|
| | | *Case-control study*—Report numbers in each exposure category, or summary measures of exposure | 17–22; Tables 2–3 | Adjusted prevalence, within-group changes, DIDs, 95% CIs, unadjusted P values, and BH-adjusted P values are reported. Unadjusted analyses are reported in eTable 5. |
| | | *Cross-sectional study*—Report numbers of outcome events or summary measures | 7–8 | Category boundaries for age and equivalized household income are reported in the Methods. |
| Main results | 16 | (*a*) Give unadjusted estimates and, if applicable, confounder-adjusted estimates and their precision (eg, 95% confidence interval). Make clear which confounders were adjusted for and why they were included | 17–22 | Absolute adjusted prevalences and absolute percentage-point changes are reported in Tables 2 and 3. |
| | | (*b*) Report category boundaries when continuous variables were categorized | 7 | Numbers are reported for baseline respondents, exclusions, valid baseline respondents, follow-up completers/noncompleters, age exclusions, and the final analytic sample. |
| | | (*c*) If relevant, consider translating estimates of relative risk into absolute risk for a meaningful time period | 7 | Reasons for prespecified exclusions are reported; the manuscript also states that individual reasons for follow-up nonparticipation were unavailable. |
| | | | | |
| Other analyses | 17 | Report other analyses done—eg analyses of subgroups and interactions, and sensitivity analyses | 13–14, 23 | Sensitivity and secondary analyses are described in the Methods and reported in the Results, with detailed results in eTables 7–11 and eFigures 1–2. |
| **Discussion** | | | | |
| Key results | 18 | Summarise key results with reference to study objectives | 24 | The Discussion summarizes the increase in telemedicine adoption, widening age-related gaps, the unemployment finding, and null DIDs for education, urbanicity, and income. |
| Limitations | 19 | Discuss limitations of the study, taking into account sources of potential bias or imprecision. Discuss both direction and magnitude of any potential bias | 26–27 | Limitations include residual confounding, lack of validated digital-literacy measures, differing outcome recall structures, attrition, exclusion of adults >75 years, modality heterogeneity, and internet-survey sampling. |
| Interpretation | 20 | Give a cautious overall interpretation of results considering objectives, limitations, multiplicity of analyses, results from similar studies, and other relevant evidence | 24–27 | The Discussion provides a cautious interpretation in relation to the inverse equity hypothesis, prior studies, sensitivity analyses, precision, and study limitations. |
| Generalisability | 21 | Discuss the generalisability (external validity) of the study results | 26–27 | Generalizability limitations are discussed for adults older than 75 years and individuals without internet access. |
| **Other information** | | | | |
| Funding | 22 | Give the source of funding and the role of the funders for the | 28 | Funding sources are reported, together with the statement that the content is |

| | | |
|---|---|---|
| | present study and, if applicable, for the original study on which the present article is based | the authors' responsibility and does not necessarily represent the funders' official views. |

*Give information separately for cases and controls in case-control studies and, if applicable, for exposed and unexposed groups in cohort and cross-sectional studies.

**Note:** An Explanation and Elaboration article discusses each checklist item and gives methodological background and published examples of transparent reporting. The STROBE checklist is best used in conjunction with this article (freely available on the Web sites of PLoS Medicine at http://www.plosmedicine.org/, Annals of Internal Medicine at http://www.annals.org/, and Epidemiology at http://www.epidem.com/). Information on the STROBE Initiative is available at www.strobe-statement.org.
