# Supplementary material for: Trends in Age and Socioeconomic Inequalities in Telemedicine Adoption in Japan, 2020-2024: Longitudinal Panel Study
Source: J Med Internet Res. 2026 Oct 8;28:e99994. doi: 10.2196/99994 (PMC13691474; doi:10.2196/99994)
Supplement: Supplementary file 2 [file jmir_v28i1e99994_app2.docx]

**Appendix**

**Supplementary Methods, Tables, and Figures**

*Trends in Age and Socioeconomic Inequalities in Telemedicine Adoption in Japan, 2020-2024: A Longitudinal Panel Study*

## Contents

Methods S1. Additional outcome, weighting, and sensitivity-analysis methods

Table S1. Management of data quality

Table S2. Harmonization of telemedicine responses and carry-forward classification

Table S3. Inverse probability weight, analytic-inclusion propensity, and trimming diagnostics

Table S4. Unweighted sample sizes and events and weighted outcome prevalence by group and survey wave

Table S5. Crude and weighted-unadjusted analyses

Table S6. Minimum detectable effects for the primary probability-scale difference-in-differences estimates

Table S7. Sensitivity analyses for age and socioeconomic inequalities in telemedicine adoption

Table S8. Continuous-age and age 65-year cutoff analyses

Table S9. Missing-income assessment and income sensitivity analyses

Table S10. Composition and refined analysis of the baseline unemployed category

Table S11. Secondary analysis of past-year telemedicine use in 2024 among participants who were never users in 2020

Figure S1. Adjusted prevalence of telemedicine adoption by continuous age

Figure S2. Adjusted change in telemedicine adoption by continuous age

# Methods S1

## Outcome harmonization and alternative definitions

The primary outcome was cumulative telemedicine adoption (ever use) at each survey wave. In August–September 2020, participants were asked whether they had experienced telemedicine since April 2020 and selected one of three responses: (1) first-time use, (2) previous use, or (3) no use. In December 2024–January 2025, participants selected one of five responses regarding telemedicine use: (1) first use within the most recent 2 months; (2) first use within the past year; (3) use within the past year but not for the first time; (4) no use within the past year but use more than 1 year earlier; or (5) no previous use. The 2020 first-time-use and previous-use responses were classified as ever use. The first four 2024 responses were classified as ever use. To preserve the cumulative interpretation of ever use, participants who reported ever use in 2020 were classified as ever users in 2024 even when their 2024 response was inconsistent with earlier use (the carry-forward rule).

Because the 2020 item did not identify use during a common 12-month recall window, an identical past-year outcome could not be reconstructed at both waves. To assess whether the primary findings depended on outcome classification, we conducted two sensitivity analyses: (1) an analysis in which telemedicine use at each wave was classified solely from the response reported at that wave, without carrying 2020 ever-use status forward to 2024; and (2) a transition analysis restricted to participants who were never users in 2020, with new adoption by 2024 defined from the reported 2024 response without carry-forward. Separately, because past-year use reflects recent telemedicine utilization rather than cumulative adoption, we conducted a secondary analysis restricted to participants who were never users in 2020, with past-year telemedicine use in 2024 defined by response options 1–3. For the analysis of past-year use among 2020 never users, weighted logistic regression models were fitted separately for age group, educational attainment, urbanicity, household income, and occupational class using the same covariates as the corresponding primary models, and adjusted probabilities and between-group risk differences were estimated using predictive margins (**eTable 11**). Cross-tabulations of the original responses and age-specific carry-forward and transition counts are reported in **eTable 2**.

## Inverse probability weighting

The final inverse probability weight was the product of a baseline sampling weight and an inverse probability-of-analytic-inclusion weight. The baseline sampling weight aligned the 2020 internet panel with the 2016 Comprehensive Survey of Living Conditions using demographic, socioeconomic, lifestyle, and health characteristics. Among all 28,000 baseline respondents, the analytic-inclusion model estimated the probability of inclusion in the final longitudinal analytic sample (10,818 participants), thereby accounting jointly for follow-up nonparticipation and prespecified analytic exclusions. The model included sex, age group, educational attainment, equivalized household-income category, occupational class, marital status, household-size category, smoking status, walking disability, hypertension, diabetes, asthma, coronary artery disease, stroke, chronic obstructive pulmonary disease, cancer, psychological disorder, and baseline telemedicine adoption. The inverse of the predicted analytic-inclusion probability was normalized to a mean of 1 among included participants. The baseline sampling and normalized analytic-inclusion weights were multiplied, and the resulting final weight was again normalized to a mean of 1.

We summarized the mean, standard deviation, minimum, selected percentiles, maximum, coefficient of variation (standard deviation divided by the mean), and effective sample size for each component and final weight. Effective sample size was calculated as (sum of weights)^2 divided by the sum of squared weights. Positivity was assessed in the 28,000-person source population by summarizing the predicted analytic-inclusion probabilities and the numbers with probabilities below 0.05, 0.10, and 0.20. As a sensitivity analysis, the final weight was winsorized at its 1st and 99th percentiles and renormalized to a mean of 1. Weight and positivity diagnostics are reported in **eTable 3**.

## Primary models and probability-scale inference

Separate repeated-observation models were fitted for age group, educational attainment, urbanicity, equivalized household income, and occupational class. Each weighted logistic regression model included the exposure, survey wave, and exposure-by-wave interaction. Fully adjusted models included sex, the other age and socioeconomic measures, marital status, household size, smoking status, walking disability, hypertension, diabetes, asthma, coronary artery disease, stroke, chronic obstructive pulmonary disease, cancer, and psychological disorder. All covariates were measured at baseline. Standard errors in the primary analysis were clustered by prefecture (47 clusters).

After each model, predictive margins were used to estimate adjusted prevalence by exposure group and wave. Within-group change was the 2024 margin minus the 2020 margin. The probability-scale difference-in-differences (DID) was the difference between that change and the corresponding change in the prespecified reference group. Confidence intervals and P values were calculated directly for the probability-scale linear contrasts of predictive margins using the delta method; they were not taken from the logit-scale interaction coefficients.

## Model-specification sensitivity analyses

For all exposures, we repeated the analysis using (1) an unweighted fully adjusted logistic model; (2) a minimally adjusted weighted logistic model; (3) a fully adjusted weighted linear probability model; (4) a fully adjusted weighted logistic model with standard errors clustered at the individual rather than prefecture level; and (5) the fully adjusted weighted logistic model with the winsorized final weight. For the minimally adjusted models, the age analysis adjusted for sex only, whereas each socioeconomic analysis adjusted for sex and age group. Results are reported in **eTable 7**.

## Sensitivity analysis with additional adjustment for digital access

As a sensitivity analysis, we repeated the fully adjusted analyses for age group, educational attainment, urbanicity, household income, and occupational class after further adjustment for baseline ownership of a smartphone, tablet, and personal computer and for home internet access as proxies for digital access. Results are reported in **eTable 7**.

## Continuous-age and age 65-year cutoff analyses

To assess whether the categorical age findings depended on cut points, baseline age was modeled continuously using a restricted cubic spline with knots at ages 25, 47, 62, and 75 years (the weighted 5th, 35th, 65th, and 95th percentiles). The spline terms and their interactions with survey wave replaced the categorical age terms; all other covariates and weighting and clustering procedures matched the primary model. Adjusted prevalence and within-age change were estimated at each integer age from 18 through 75 years, with selected values reported in **eTable 8** and the full curves shown in **eFigures 1 and 2**. We also repeated the model using a binary age variable (18-64 vs 65-75 years).

## Income missingness and sensitivity analyses

The primary analysis retained participants without reported household income as a separate category. Robustness was assessed in a complete-case analysis excluding missing income; a multiple-imputation analysis; and a continuous-income analysis. For multiple imputation, the 3-level income category (high, medium, or low) was imputed using ordered logistic regression with 20 imputations. The imputation model included sex, age group, educational attainment, urbanicity, occupational class, marital status, household size, smoking, walking disability, a comorbidity count, baseline telemedicine adoption, and 2024 telemedicine adoption. DID estimates were combined using Rubin rules. For the continuous analysis, log(equivalized household income + 1) was modeled continuously, and changes were contrasted at the weighted 25th and 75th percentiles of equivalized household income. Results are reported in **eTable 9**.

## Crude and unadjusted analyses

We additionally estimated unweighted crude and weighted unadjusted DIDs from models containing only the exposure, survey wave, and their interaction. Results are reported in **eTable 5**.

## Refined baseline occupational categories

The baseline unemployed category was separated into students (including preparatory-school students), retired participants, homemakers, and other participants not employed. The four employed categories were retained, producing an 8-level occupational variable. We repeated the fully adjusted weighted repeated-wave model using the refined 8-level occupational variable and upper non-manual workers as the reference. For the analysis of new adoption among 2020 never users, we fitted a weighted logistic regression model restricted to participants who reported no telemedicine use in 2020, using the same covariates as in the primary occupational model. Adjusted new-adoption risks and risk differences versus upper non-manual workers were estimated using predictive margins. Occupational status was baseline-defined; transitions in employment after 2020 were not modeled. Results are reported in **eTable 10**.

## Sparse cells and model convergence

We tabulated unweighted outcome events for every exposure-group-by-wave cell and for the refined occupational analysis and documented model convergence. No refined occupational cell contained zero events, and both the repeated-wave and transition models converged. Robustness was evaluated with simplified adjustment, unweighted models, a linear probability model, and cell-level event counts. Unweighted sample sizes and outcome events for the primary exposure-group-by-wave cells are reported in **eTable 4**.

## Precision and multiplicity

For each of the 15 nonreference DIDs, an approximate minimum detectable effect (MDE) for 80% power at a 2-sided alpha of .05 was calculated as (1.96 + 0.84) multiplied by the DID standard error (**eTable 6**). To account for multiple comparisons, Benjamini-Hochberg false-discovery-rate-adjusted P values were calculated across the 15 nonreference contrasts in the primary analyses. All analyses used Stata/MP version 18.0 (StataCorp). Intermediate JACSIS waves were unavailable for this analytic cohort; therefore, a 2021-2023 trend or placebo analysis was not performed.

Table S1. Management of data quality.

| **Criterion** | **Exclusion rule** |
| --- | --- |
| A | Incorrect response to the attention-check item: 'Please choose the second from the bottom of the following options: A, B, C, D, E.' The correct response was D. |
| B | Reported 'almost every day' or 'several times per week' use for all nine substances for which such a combined pattern was considered implausible or inconsistent: alcohol; sleeping pills or anti-anxiety drugs; prescribed narcotics for cancer pain; prescribed narcotics for noncancer pain; nonprescribed narcotics; inhaled organic solvents; illegal herbs or magic mushrooms; cannabis; and methamphetamine, cocaine, or heroin. |
| C | Reported currently having, with or without treatment, every one of 16 chronic conditions: hypertension, diabetes, asthma, bronchitis or pneumonia, atopic dermatitis, periodontal disease, caries, otitis media, angina pectoris, myocardial infarction, stroke, chronic obstructive pulmonary disease, cancer or malignant tumor, chronic pain, depression, and a mental disorder other than depression. |

*Respondents meeting any criterion were classified as providing potentially unreliable or inconsistent responses and were excluded before the longitudinal analytic sample was constructed.*

Table S2. Harmonization of telemedicine responses and carry-forward classification.

| **Panel A. Cross-tabulation of the original 2020 and 2024 responses** | | | | | |
| --- | --- | --- | --- | --- | --- |
| **2020 response** | **2024 opt 1** | **2024 opt 2** | **2024 opt 3** | **2024 opt 4** | **2024 opt 5** |
| 2020 option 1: first use | 1 [0.00%] | 5 [0.04%] | 19 [0.20%] | 48 [0.34%] | 111 [0.86%] |
| 2020 option 2: previous use | 1 [0.01%] | 6 [0.11%] | 14 [0.16%] | 14 [0.16%] | 63 [0.88%] |
| 2020 option 3: no previous use | 23 [0.18%] | 89 [1.03%] | 137 [1.52%] | 227 [2.50%] | 10,060 [91.98%] |

*Cells show unweighted n [weighted percentage of the full analytic sample]. 2024 options were: 1, first use within the most recent 2 months; 2, first use within the past year; 3, use within the past year but not for the first time; 4, use more than 1 year ago but not within the past year; and 5, no previous use. The primary 2024 ever-use outcome included options 1-4 and additionally carried forward 2020 ever users whose 2024 response was inconsistent with previous use.*

| **Panel B. Carry-forward classification and new adoption by age** | | | | | |
| --- | --- | --- | --- | --- | --- |
| **Age, y** | **2020 ever users, n** | **Carried forward, n** | **Carry-forward among 2024 users, weighted %** | **New adopters, n** | **New adoption among 2020 never users, weighted %** |
| 18–29 | 45 | 29 | 19.7 | 69 | 11.1 |
| 30–39 | 47 | 17 | 14.1 | 100 | 8.1 |
| 40–49 | 60 | 35 | 20.1 | 135 | 5.8 |
| 50–59 | 47 | 29 | 22.7 | 103 | 4.4 |
| 60–69 | 43 | 32 | 23.8 | 46 | 2.4 |
| 70–75 | 40 | 32 | 58.6 | 23 | 1.2 |

*New adopters were participants classified as never users in 2020 who reported use by 2024. Percentages use the final normalized inverse probability weight. Age was measured in 2020.*

Table S3. Inverse probability weight, analytic-inclusion propensity, and trimming diagnostics.

| **Panel A. Weight distributions** | | | | | | | | | | |
| --- | --- | --- | --- | --- | --- | --- | --- | --- | --- | --- |
| **Weight** | **N** | **Mean** | **SD** | **Min** | **P1** | **P50** | **P99** | **Max** | **CV** | **ESS** |
| Baseline sampling weight | 10,818 | 1.000 | 1.253 | 0.070 | 0.110 | 0.555 | 5.650 | 19.001 | 1.253 | 4,209 |
| Analytic-inclusion weight | 10,818 | 1.000 | 0.406 | 0.570 | 0.638 | 0.865 | 2.574 | 4.782 | 0.406 | 9,285 |
| Final product weight | 10,818 | 1.000 | 1.417 | 0.051 | 0.082 | 0.529 | 6.962 | 27.050 | 1.417 | 3,598 |

*CV indicates coefficient of variation; ESS, effective sample size. Weight distributions are summarized among the 10,818 participants in the final analytic sample.*

| **Panel B. Predicted analytic-inclusion probability in the 28,000-person source population** | | | |
| --- | --- | --- | --- |
| **Diagnostic** | | **Value** | |
| Source population, n | | 28,000 | |
| Included in final analytic sample, n | | 10,818 | |
| Mean (SD) | | 0.386 (0.125) | |
| Minimum; P1; P5 | | 0.057; 0.122; 0.164 | |
| P10; P25; P50 | | 0.192; 0.294; 0.415 | |
| P75; P90; P95; P99; maximum | | 0.482; 0.531; 0.555; 0.602; 0.679 | |
| Probability <0.05, n (%) | | 0 (0.00) | |
| Probability <0.10, n (%) | | 74 (0.26) | |
| Probability <0.20, n (%) | | 3,244 (11.59) | |
| **Panel C. Distribution across predicted-probability ranges** | | | |
| **Predicted probability** | **n** | | **%** |
| 0.05 to <0.10 | 74 | | 0.26 |
| 0.10 to <0.20 | 3,170 | | 11.32 |
| 0.20 to <0.50 | 19,484 | | 69.59 |
| 0.50 to <0.80 | 5,272 | | 18.83 |

*No participant had a predicted analytic-inclusion probability below 0.05; 74 (0.26%) had a probability below 0.10. In the trimming sensitivity analysis, the final weight was winsorized at P1=0.082 and P99=6.962 (107 observations below P1 and 108 above P99) and then renormalized to mean 1.*

Table S4. Unweighted sample sizes and events and weighted outcome prevalence by group and survey wave.

| **Exposure** | **Group** | **Participants, n** | **2020 events, n** | **2020 weighted prevalence, %** | **2024 events, n** | **2024 weighted prevalence, %** |
| --- | --- | --- | --- | --- | --- | --- |
| Age, y | 18–29 | 913 | 45 | 5.5 | 114 | 16.0 |
|  | 30–39 | 1,240 | 47 | 3.8 | 147 | 11.6 |
|  | 40–49 | 2,185 | 60 | 2.3 | 195 | 7.9 |
|  | 50–59 | 2,165 | 47 | 2.3 | 150 | 6.6 |
|  | 60–69 | 2,376 | 43 | 1.1 | 89 | 3.5 |
|  | 70–75 | 1,939 | 40 | 2.0 | 63 | 3.1 |
| Educational attainment | University or higher | 5,314 | 183 | 5.1 | 453 | 11.4 |
|  | 2-year college | 2,346 | 48 | 2.1 | 131 | 7.6 |
|  | High school or lower | 3,158 | 51 | 1.9 | 174 | 6.5 |
| Urbanicity | Urban | 7,542 | 216 | 3.3 | 548 | 8.1 |
|  | Rural | 3,276 | 66 | 1.9 | 210 | 7.9 |
| Equivalized household income | High (≥4.30 million JPY) | 2,882 | 106 | 4.2 | 280 | 10.2 |
|  | Medium (2.50-4.30 million JPY) | 2,554 | 50 | 1.7 | 168 | 8.0 |
|  | Low (<2.50 million JPY) | 3,265 | 84 | 3.6 | 201 | 8.4 |
|  | Not answered | 2,117 | 42 | 1.5 | 109 | 5.4 |
| Occupational class | Upper non-manual | 2,800 | 105 | 4.8 | 295 | 11.9 |
|  | Lower non-manual | 1,767 | 39 | 2.6 | 132 | 8.2 |
|  | Manual | 1,295 | 24 | 2.3 | 102 | 12.7 |
|  | Self-employed | 824 | 28 | 4.7 | 56 | 8.6 |
|  | Unemployed | 4,132 | 86 | 1.6 | 173 | 3.9 |

*The same participants contributed to both waves. Counts and events are unweighted; prevalence estimates use the final normalized inverse probability weight and are unadjusted. The primary outcome applies the carry-forward rule.*

Table S5. Crude and weighted-unadjusted analyses.

| **Domain** | **Comparison with reference** | **Unweighted crude DID, pp (95% CI)** | **P value** | **Weighted unadjusted DID, pp (95% CI)** | **P value** |
| --- | --- | --- | --- | --- | --- |
| Age | 30–39 years | +0.5 (-1.9 to +2.9) | .683 | -2.6 (-8.1 to +2.8) | .343 |
|  | 40–49 years | -1.4 (-3.4 to +0.7) | .184 | -4.8 (-9.9 to +0.2) | .061 |
|  | 50–59 years | -2.8 (-5.0 to -0.6) | .011 | -6.1 (-10.9 to -1.3) | .012 |
|  | 60–69 years | -5.6 (-7.6 to -3.6) | <.001 | -8.1 (-13.0 to -3.1) | .001 |
|  | 70–75 years | -6.4 (-8.1 to -4.7) | <.001 | -9.3 (-14.0 to -4.6) | <.001 |
| Education | 2-year college | -1.5 (-2.6 to -0.5) | .004 | -0.9 (-3.6 to +1.8) | .530 |
|  | High school or lower | -1.2 (-2.1 to -0.3) | .013 | -1.7 (-3.6 to +0.2) | .081 |
| Urbanicity | Rural | -0.0 (-0.9 to +0.9) | .989 | +1.1 (-0.7 to +2.9) | .240 |
| Income | Medium | -1.4 (-2.8 to -0.0) | .042 | +0.2 (-2.6 to +3.1) | .873 |
|  | Low | -2.5 (-3.6 to -1.3) | <.001 | -1.1 (-2.9 to +0.7) | .215 |
|  | Not answered | -2.9 (-4.1 to -1.6) | <.001 | -2.2 (-4.5 to +0.2) | .070 |
| Occupation | Lower non-manual workers | -1.5 (-2.7 to -0.3) | .014 | -1.4 (-4.0 to +1.1) | .276 |
|  | Manual workers | -0.8 (-2.9 to +1.4) | .488 | +3.4 (-2.5 to +9.2) | .257 |
|  | Self-employed | -3.4 (-5.0 to -1.7) | <.001 | -3.2 (-5.3 to -1.0) | .004 |
|  | Unemployed | -4.7 (-5.8 to -3.6) | <.001 | -4.7 (-6.5 to -2.9) | <.001 |

*Crude and unadjusted models included the exposure, survey wave, and exposure-by-wave interaction only. Reference groups were age 18-29 years, university education or higher, urban residence, high income, and upper non-manual workers. DID indicates difference-in-differences; pp, percentage points.*

Table S6. Minimum detectable effects for the primary probability-scale difference-in-differences estimates.

| **Domain** | **Comparison with reference** | **DID, pp (95% CI)** | **MDE, pp** |
| --- | --- | --- | --- |
| Education | 2-year college | +0.5 (-2.1 to +3.1) | 3.7 |
|  | High school or lower | -0.4 (-2.1 to +1.4) | 2.6 |
| Age | 30–39 years | -5.2 (-11.8 to +1.4) | 9.5 |
|  | 40–49 years | -7.7 (-14.2 to -1.1) | 9.4 |
|  | 50–59 years | -9.1 (-15.3 to -2.9) | 8.9 |
|  | 60–69 years | -10.5 (-16.7 to -4.3) | 8.9 |
|  | 70–75 years | -12.1 (-18.2 to -5.9) | 8.8 |
| Occupation | Lower non-manual workers | -0.2 (-2.6 to +2.2) | 3.5 |
|  | Manual workers | +3.7 (-1.2 to +8.6) | 7.0 |
|  | Self-employed | -1.6 (-3.6 to +0.5) | 2.9 |
|  | Unemployed | -2.9 (-4.6 to -1.2) | 2.4 |
| Income | Medium | +0.3 (-1.9 to +2.5) | 3.1 |
|  | Low | +0.2 (-1.5 to +1.9) | 2.4 |
|  | Not answered | -0.7 (-3.1 to +1.6) | 3.4 |
| Urbanicity | Rural | +1.6 (-0.1 to +3.3) | 2.4 |

*Reference groups were age 18-29 years, university education or higher, urban residence, high income, and upper non-manual workers. DID indicates difference-in-differences; pp, percentage points; MDE, approximate minimum detectable DID for 80% power at 2-sided alpha=.05.*

Table S7. Sensitivity analyses for age and socioeconomic inequalities in telemedicine adoption.

| **Panel A. Model-specification sensitivity analyses** | | | | | | | |
| --- | --- | --- | --- | --- | --- | --- | --- |
| **Domain** | **Comparison with reference** | **Primary, DID pp (95% CI)** | **Unweighted fully adjusted, DID pp (95% CI)** | **Minimally adjusted, DID pp (95% CI)** | **Linear probability model, DID pp (95% CI)** | **Person-clustered SE, DID pp (95% CI)** | **Trimmed weight, DID pp (95% CI)** |
| Age | 30–39 years | -5.2 (-11.8 to +1.4); adjusted P=.235 | -0.8 (-3.7 to +2.2); adjusted P=.706 | -2.9 (-8.5 to +2.6); adjusted P=.505 | -2.6 (-8.2 to +3.0); adjusted P=.402 | -5.2 (-11.2 to +0.8); adjusted P=.195 | -2.8 (-7.2 to +1.6); adjusted P=.403 |
|  | 40–49 years | -7.7 (-14.2 to -1.1); adjusted P=.066 | -3.2 (-5.7 to -0.8); adjusted P=.023 | -5.1 (-10.2 to +0.1); adjusted P=.171 | -4.8 (-10.0 to +0.4); adjusted P=.163 | -7.7 (-13.6 to -1.7); adjusted P=.033 | -5.5 (-10.0 to -1.0); adjusted P=.054 |
|  | 50–59 years | -9.1 (-15.3 to -2.9); adjusted P=.016 | -5.2 (-7.5 to -2.8); adjusted P=.002 | -6.3 (-11.3 to -1.3); adjusted P=.049 | -6.1 (-11.1 to -1.2); adjusted P=.048 | -9.1 (-14.9 to -3.3); adjusted P=.008 | -7.0 (-11.3 to -2.8); adjusted P=.005 |
|  | 60–69 years | -10.5 (-16.7 to -4.3); adjusted P=.004 | -7.6 (-9.9 to -5.3); adjusted P=.002 | -8.2 (-13.3 to -3.0); adjusted P=.010 | -8.1 (-13.1 to -3.0); adjusted P=.015 | -10.5 (-16.2 to -4.8); adjusted P=.003 | -8.6 (-13.0 to -4.1); adjusted P=.004 |
|  | 70–75 years | -12.1 (-18.2 to -5.9); adjusted P=.002 | -8.3 (-10.5 to -6.1); adjusted P=.002 | -9.5 (-14.4 to -4.6); adjusted P=.004 | -9.3 (-14.1 to -4.5); adjusted P=.004 | -12.1 (-17.8 to -6.4); adjusted P=.003 | -10.1 (-14.3 to -5.9); adjusted P=.004 |
| Education | 2-year college | +0.5 (-2.1 to +3.1); adjusted P=.862 | -0.7 (-1.7 to +0.3); adjusted P=.257 | +0.3 (-2.5 to +3.1); adjusted P=.855 | -0.9 (-3.7 to +1.9); adjusted P=.571 | +0.5 (-1.8 to +2.8); adjusted P=.863 | -0.0 (-2.1 to +2.1); adjusted P=.970 |
|  | High school or lower | -0.4 (-2.1 to +1.4); adjusted P=.862 | -0.1 (-1.1 to +0.9); adjusted P=.832 | -0.6 (-2.5 to +1.3); adjusted P=.676 | -1.7 (-3.7 to +0.3); adjusted P=.165 | -0.4 (-2.1 to +1.3); adjusted P=.863 | -0.8 (-2.1 to +0.6); adjusted P=.419 |
| Urbanicity | Rural | +1.6 (-0.1 to +3.3); adjusted P=.170 | +0.2 (-0.6 to +1.0); adjusted P=.706 | +1.7 (-0.1 to +3.6); adjusted P=.175 | +1.1 (-0.8 to +3.0); adjusted P=.353 | +1.6 (-0.4 to +3.6); adjusted P=.229 | +1.0 (-0.3 to +2.3); adjusted P=.296 |
| Income | Medium | +0.3 (-1.9 to +2.5); adjusted P=.862 | -0.9 (-2.2 to +0.4); adjusted P=.257 | +0.2 (-2.3 to +2.8); adjusted P=.855 | +0.2 (-2.7 to +3.2); adjusted P=.874 | +0.3 (-2.0 to +2.6); adjusted P=.872 | -0.5 (-2.1 to +1.2); adjusted P=.731 |
|  | Low | +0.2 (-1.5 to +1.9); adjusted P=.862 | -1.3 (-2.6 to -0.1); adjusted P=.056 | -0.3 (-2.1 to +1.5); adjusted P=.855 | -1.1 (-3.0 to +0.7); adjusted P=.353 | +0.2 (-1.7 to +2.1); adjusted P=.872 | +0.3 (-1.4 to +2.0); adjusted P=.853 |
|  | Not answered | -0.7 (-3.1 to +1.6); adjusted P=.810 | -1.8 (-3.1 to -0.5); adjusted P=.018 | -1.6 (-3.8 to +0.5); adjusted P=.268 | -2.2 (-4.5 to +0.2); adjusted P=.163 | -0.7 (-3.1 to +1.6); adjusted P=.791 | -1.0 (-2.8 to +0.8); adjusted P=.419 |
| Occupation | Lower non-manual workers | -0.2 (-2.6 to +2.2); adjusted P=.881 | -0.4 (-1.4 to +0.6); adjusted P=.638 | -0.9 (-3.2 to +1.5); adjusted P=.652 | -1.4 (-4.1 to +1.2); adjusted P=.353 | -0.2 (-2.4 to +2.1); adjusted P=.872 | -0.2 (-2.4 to +2.0); adjusted P=.921 |
|  | Manual workers | +3.7 (-1.2 to +8.6); adjusted P=.235 | -0.2 (-2.0 to +1.6); adjusted P=.832 | +2.3 (-2.6 to +7.1); adjusted P=.534 | +3.4 (-2.6 to +9.4); adjusted P=.353 | +3.7 (-0.5 to +7.9); adjusted P=.195 | +1.6 (-1.5 to +4.8); adjusted P=.423 |
|  | Self-employed | -1.6 (-3.6 to +0.5); adjusted P=.235 | -2.0 (-3.5 to -0.4); adjusted P=.028 | -1.9 (-4.1 to +0.3); adjusted P=.186 | -3.2 (-5.4 to -1.0); adjusted P=.023 | -1.6 (-3.9 to +0.7); adjusted P=.292 | -1.7 (-3.7 to +0.3); adjusted P=.258 |
|  | Unemployed | -2.9 (-4.6 to -1.2); adjusted P=.004 | -2.9 (-3.8 to -1.9); adjusted P=.002 | -3.2 (-4.9 to -1.5); adjusted P=.004 | -4.7 (-6.5 to -2.8); adjusted P=.004 | -2.9 (-4.6 to -1.2); adjusted P=.003 | -2.8 (-4.5 to -1.0); adjusted P=.008 |

*Cells show probability-scale difference-in-differences (DIDs) in percentage points (95% CI) and Benjamini–Hochberg false-discovery-rate-adjusted P value across the 15 nonreference contrasts within each model specification. The primary model was a fully adjusted weighted logistic model with prefecture-clustered standard errors. For the minimally adjusted models, the age analysis adjusted for sex only, whereas each socioeconomic analysis adjusted for sex and age group. The trimmed-weight model used the winsorized final weight.*

Table S7. Sensitivity analyses for age and socioeconomic inequalities in telemedicine adoption (continued).

| **Panel B. Analyses using alternative outcome classifications** | | | | |
| --- | --- | --- | --- | --- |
| **Exposure** | **Comparison** | **Primary cumulative adoption, DID pp (95% CI)** | **No carry-forward, DID pp (95% CI)** | **New adoption among 2020 never users, RD pp (95% CI)** |
| Age group | 30–39 vs 18–29 years | -5.2 (-11.8 to +1.4); adjusted P=.235 | -2.9 (-10.4 to +4.6); adjusted P=.525 | -5.2 (-11.2 to +0.8); adjusted P=.198 |
|  | 40–49 vs 18–29 years | -7.7 (-14.2 to -1.1); adjusted P=.066 | -5.1 (-12.6 to +2.3); adjusted P=.377 | -7.1 (-13.4 to -0.8); adjusted P=.081 |
|  | 50–59 vs 18–29 years | -9.1 (-15.3 to -2.9); adjusted P=.016 | -6.4 (-13.4 to +0.7); adjusted P=.232 | -8.1 (-14.4 to -1.9); adjusted P=.041 |
|  | 60–69 vs 18–29 years | -10.5 (-16.7 to -4.3); adjusted P=.004 | -7.2 (-14.3 to -0.2); adjusted P=.176 | -9.4 (-15.6 to -3.2); adjusted P=.015 |
|  | 70–75 vs 18–29 years | -12.1 (-18.2 to -5.9); adjusted P=.002 | -9.6 (-16.6 to -2.5); adjusted P=.116 | -10.8 (-17.0 to -4.7); adjusted P=.008 |
| Educational attainment | 2-year college vs university or higher | +0.5 (-2.1 to +3.1); adjusted P=.862 | +1.7 (-1.3 to +4.7); adjusted P=.380 | -0.2 (-2.5 to +2.2); adjusted P=.894 |
|  | High school or lower vs university or higher | -0.4 (-2.1 to +1.4); adjusted P=.862 | +1.0 (-1.3 to +3.3); adjusted P=.490 | -1.0 (-2.6 to +0.7); adjusted P=.359 |
| Urbanicity | Rural vs urban | +1.6 (-0.1 to +3.3); adjusted P=.170 | +2.1 (+0.0 to +4.1); adjusted P=.176 | +1.4 (-0.1 to +2.8); adjusted P=.187 |
| Household income | Medium vs high | +0.3 (-1.9 to +2.5); adjusted P=.862 | +1.6 (-1.0 to +4.2); adjusted P=.380 | -0.4 (-2.2 to +1.4); adjusted P=.716 |
|  | Low vs high | +0.2 (-1.5 to +1.9); adjusted P=.862 | -0.5 (-2.9 to +2.0); adjusted P=.758 | +0.4 (-1.5 to +2.3); adjusted P=.716 |
|  | Not answered vs high | -0.7 (-3.1 to +1.6); adjusted P=.810 | +0.0 (-2.9 to +3.0); adjusted P=.986 | -1.5 (-3.8 to +0.9); adjusted P=.359 |
| Occupational class | Lower non-manual vs upper non-manual | -0.2 (-2.6 to +2.2); adjusted P=.881 | +1.2 (-1.0 to +3.5); adjusted P=.380 | -0.7 (-3.1 to +1.6); adjusted P=.662 |
|  | Manual vs upper non-manual | +3.7 (-1.2 to +8.6); adjusted P=.235 | +5.0 (+0.1 to +9.9); adjusted P=.176 | +2.5 (-1.9 to +7.0); adjusted P=.359 |
|  | Self-employed vs upper non-manual | -1.6 (-3.6 to +0.5); adjusted P=.235 | -3.4 (-7.6 to +0.7); adjusted P=.263 | -1.3 (-3.5 to +0.9); adjusted P=.359 |
|  | Unemployed vs upper non-manual | -2.9 (-4.6 to -1.2); adjusted P=.004 | -1.4 (-3.6 to +0.8); adjusted P=.377 | -2.9 (-4.8 to -1.0); adjusted P=.015 |

*The primary and no-carry-forward estimates are probability-scale difference-in-differences (DIDs) comparing changes from 2020 to 2024 with the reference category. The new-adoption analysis was restricted to participants who had never used telemedicine in 2020 and reports adjusted risk differences (RDs) in 2024. All models used the same covariate adjustment set as the primary analyses and inverse probability weighting, with standard errors clustered by prefecture. Benjamini–Hochberg false-discovery-rate-adjusted P values were calculated across the 15 nonreference contrasts within each analysis.*

Table S7. Sensitivity analyses for age and socioeconomic inequalities in telemedicine adoption (continued).

| **Panel C. Sensitivity analysis with additional adjustment for digital-access proxies** | | | |
| --- | --- | --- | --- |
| **Exposure** | **Comparison** | **Primary cumulative adoption, DID pp (95% CI)** | **Additionally adjusted for digital-access proxies, DID pp (95% CI)** |
| Age group | 30–39 vs 18–29 years | -5.2 (-11.8 to +1.4); adjusted P=.235 | −5.5 (−12.3 to +1.3); adjusted P=.194 |
|  | 40–49 vs 18–29 years | -7.7 (-14.2 to -1.1); adjusted P=.066 | −7.8 (−14.5 to −1.1); adjusted P=.065 |
|  | 50–59 vs 18–29 years | -9.1 (-15.3 to -2.9); adjusted P=.016 | −9.2 (−15.6 to −2.8); adjusted P=.018 |
|  | 60–69 vs 18–29 years | -10.5 (-16.7 to -4.3); adjusted P=.004 | −10.5 (−16.9 to −4.1); adjusted P=.009 |
|  | 70–75 vs 18–29 years | -12.1 (-18.2 to -5.9); adjusted P=.002 | −12.2 (−18.5 to −5.9); adjusted P=.002 |
| Educational attainment | 2-year college vs university or higher | +0.5 (-2.1 to +3.1); adjusted P=.862 | +0.4 (−2.2 to +3.1); adjusted P=.840 |
|  | High school or lower vs university or higher | -0.4 (-2.1 to +1.4); adjusted P=.862 | −0.4 (−2.1 to +1.4); adjusted P=.840 |
| Urbanicity | Rural vs urban | +1.6 (-0.1 to +3.3); adjusted P=.170 | +1.4 (−0.3 to +3.2); adjusted P=.194 |
| Household income | Medium vs high | +0.3 (-1.9 to +2.5); adjusted P=.862 | +0.5 (−1.7 to +2.7); adjusted P=.840 |
|  | Low vs high | +0.2 (-1.5 to +1.9); adjusted P=.862 | +0.2 (−1.5 to +1.9); adjusted P=.840 |
|  | Not answered vs high | -0.7 (-3.1 to +1.6); adjusted P=.810 | −0.6 (−3.0 to +1.7); adjusted P=.840 |
| Occupational class | Lower non-manual vs upper non-manual | -0.2 (-2.6 to +2.2); adjusted P=.881 | −0.0 (−2.5 to +2.5); adjusted P=.993 |
|  | Manual vs upper non-manual | +3.7 (-1.2 to +8.6); adjusted P=.235 | +3.9 (−1.0 to +8.8); adjusted P=.194 |
|  | Self-employed vs upper non-manual | -1.6 (-3.6 to +0.5); adjusted P=.235 | −1.8 (−3.8 to +0.3); adjusted P=.194 |
|  | Unemployed vs upper non-manual | -2.9 (-4.6 to -1.2); adjusted P=.004 | −2.8 (−4.5 to −1.0); adjusted P=.009 |

*Digital-access proxies included baseline ownership of a smartphone, tablet, and personal computer and home internet access. These variables were added to the fully adjusted primary models as a sensitivity analysis. Estimates are probability-scale difference-in-differences (DIDs) in percentage points, with 95% CIs. Models used inverse probability weighting and prefecture-clustered standard errors. Benjamini–Hochberg false-discovery-rate-adjusted P values were calculated across the 15 nonreference contrasts within each analysis.*

Table S8. Continuous-age and age 65-year cutoff analyses.

| **Panel A. Restricted cubic spline estimates at selected ages** | | | | |
| --- | --- | --- | --- | --- |
| **Age in 2020, y** | **2020 adjusted prevalence, % (95% CI)** | **2024 adjusted prevalence, % (95% CI)** | **Adjusted change, pp (95% CI)** | **P value for change** |
| 20 | 8.7 (4.1 to 13.2) | 24.8 (15.7 to 33.8) | +16.1 (+7.8 to +24.3) | <.001 |
| 30 | 5.1 (3.8 to 6.5) | 15.3 (12.5 to 18.2) | +10.2 (+7.7 to +12.7) | <.001 |
| 40 | 3.0 (2.3 to 3.8) | 9.3 (7.6 to 11.1) | +6.3 (+4.9 to +7.6) | <.001 |
| 50 | 1.9 (1.3 to 2.5) | 6.2 (4.9 to 7.4) | +4.3 (+3.4 to +5.2) | <.001 |
| 60 | 1.4 (0.8 to 1.9) | 4.6 (3.6 to 5.6) | +3.2 (+2.3 to +4.1) | <.001 |
| 65 | 1.4 (1.0 to 1.9) | 4.0 (3.0 to 5.0) | +2.5 (+1.6 to +3.5) | <.001 |
| 70 | 1.7 (1.2 to 2.1) | 3.4 (2.3 to 4.5) | +1.7 (+0.9 to +2.6) | <.001 |
| 75 | 2.0 (0.9 to 3.1) | 2.9 (1.4 to 4.4) | +0.9 (+0.0 to +1.7) | .044 |

*Age was modeled with a restricted cubic spline using knots at ages 25, 47, 62, and 75 years. Values were estimated from the fully adjusted, weighted model with standard errors clustered by prefecture.*

| **Panel B. Binary age cutoff at 65 years** | | | | | |
| --- | --- | --- | --- | --- | --- |
| **Age group** | **2020 adjusted prevalence, % (95% CI)** | **2024 adjusted prevalence, % (95% CI)** | **Change, pp (95% CI)** | **DID, pp (95% CI)** | **P value** |
| Age 18–64 years | 3.0 (2.4 to 3.6) | 9.2 (8.0 to 10.5) | +6.2 (+5.2 to +7.3) | Reference |  |
| Age 65–75 years | 2.0 (1.1 to 2.8) | 3.6 (2.2 to 5.0) | +1.6 (+0.7 to +2.5) | -4.6 (-5.9 to -3.4) | <.001 |

*The 18-64-year group was the reference. DID indicates probability-scale difference-in-differences; pp, percentage points.*

Table S9. Missing-income assessment and income sensitivity analyses.

| **Panel A. Baseline characteristics according to availability of household-income data** | | | | | |
| --- | --- | --- | --- | --- | --- |
| **Characteristic** | **Category** | **Income reported, unweighted** | **Income missing, unweighted** | **Income reported, weighted** | **Income missing, weighted** |
| Age, years | Mean (SD) | 53.5 (15.2) | 52.9 (15.4) | 50.1 (16.6) | 48.4 (17.2) |
| Sex | Female | 3,802 (43.7) | 1,209 (57.1) | 47.2 | 62.6 |
|  | Male | 4,899 (56.3) | 908 (42.9) | 52.8 | 37.4 |
| Age group | 18–29 years | 706 (8.1) | 207 (9.8) | 14.7 | 19.5 |
|  | 30–39 years | 1,028 (11.8) | 212 (10.0) | 15.4 | 12.3 |
|  | 40–49 years | 1,759 (20.2) | 426 (20.1) | 19.4 | 19.6 |
|  | 50–59 years | 1,711 (19.7) | 454 (21.4) | 17.7 | 17.9 |
|  | 60–69 years | 1,899 (21.8) | 477 (22.5) | 15.4 | 16.9 |
|  | 70–75 years | 1,598 (18.4) | 341 (16.1) | 17.5 | 13.8 |
| Educational attainment | University or higher | 4,430 (50.9) | 884 (41.8) | 27.3 | 21.7 |
|  | 2-year college | 1,813 (20.8) | 533 (25.2) | 20.2 | 21.1 |
|  | High school or lower | 2,458 (28.2) | 700 (33.1) | 52.6 | 57.2 |
| Urbanicity of residence | Urban | 6,141 (70.6) | 1,401 (66.2) | 62.2 | 57.4 |
|  | Rural | 2,560 (29.4) | 716 (33.8) | 37.8 | 42.6 |
| Occupational class | Upper non-manual workers | 2,446 (28.1) | 354 (16.7) | 24.1 | 14.2 |
|  | Lower non-manual workers | 1,420 (16.3) | 347 (16.4) | 18.1 | 15.8 |
|  | Manual workers | 1,087 (12.5) | 208 (9.8) | 15.1 | 12.8 |
|  | Self-employed | 644 (7.4) | 180 (8.5) | 6.8 | 7.4 |
|  | Unemployed | 3,104 (35.7) | 1,028 (48.6) | 35.9 | 49.8 |
| Marital status | Married | 5,650 (64.9) | 1,219 (57.6) | 68.0 | 57.1 |
|  | Never married | 2,120 (24.4) | 675 (31.9) | 20.8 | 32.2 |
|  | Widowed | 319 (3.7) | 79 (3.7) | 5.5 | 4.8 |
|  | Separated | 612 (7.0) | 144 (6.8) | 5.6 | 6.0 |
| Household size | 1 | 1,828 (21.0) | 295 (13.9) | 16.7 | 11.2 |
|  | 2 | 3,289 (37.8) | 761 (35.9) | 35.5 | 33.1 |
|  | 3 | 1,857 (21.3) | 576 (27.2) | 21.9 | 27.1 |
|  | 4 | 1,225 (14.1) | 336 (15.9) | 17.4 | 19.6 |
|  | 5 or more | 502 (5.8) | 149 (7.0) | 8.4 | 9.1 |
| Smoking status | Never | 4,297 (49.4) | 1,278 (60.4) | 49.3 | 63.7 |
|  | Ever | 2,599 (29.9) | 468 (22.1) | 29.7 | 19.0 |
|  | Current | 1,805 (20.7) | 371 (17.5) | 21.1 | 17.2 |

Table S9. Missing-income assessment and income sensitivity analyses (continued).

| **Panel A (continued). Baseline health characteristics and telemedicine adoption** | | | | | |
| --- | --- | --- | --- | --- | --- |
| **Characteristic** | **Category** | **Income reported, unweighted** | **Income missing, unweighted** | **Income reported, weighted** | **Income missing, weighted** |
| Walking disability | Yes | 659 (7.6) | 192 (9.1) | 10.0 | 11.2 |
| Hypertension | Yes | 1,965 (22.6) | 401 (18.9) | 21.3 | 17.5 |
| Diabetes | Yes | 662 (7.6) | 143 (6.8) | 8.6 | 7.7 |
| Asthma | Yes | 313 (3.6) | 73 (3.4) | 4.7 | 3.9 |
| Coronary artery disease | Yes | 232 (2.7) | 34 (1.6) | 3.5 | 2.3 |
| Stroke | Yes | 130 (1.5) | 19 (0.9) | 2.5 | 1.1 |
| Chronic obstructive pulmonary disease | Yes | 110 (1.3) | 14 (0.7) | 1.8 | 0.9 |
| Cancer | Yes | 194 (2.2) | 35 (1.7) | 3.0 | 2.0 |
| Psychological disorder | Yes | 487 (5.6) | 124 (5.9) | 5.9 | 5.8 |
| Baseline telemedicine adoption | Yes | 240 (2.8) | 42 (2.0) | 3.1 | 1.5 |

*Among 10,818 participants, 8,701 reported household income and 2,117 did not. For age, values are mean (SD). For categorical variables, unweighted values are n (%) and weighted values are percentages. Percentages use the final normalized inverse probability weight. For binary health conditions, only the 'Yes' category is shown.*

| **Panel B. Income sensitivity analyses** | | | |
| --- | --- | --- | --- |
| **Analysis** | **Contrast** | **DID, pp (95% CI)** | **P value** |
| Primary missing-category analysis | Medium vs high | +0.3 (-1.9 to +2.5) | .793 |
|  | Low vs high | +0.2 (-1.5 to +1.9) | .805 |
|  | Not answered vs high | -0.7 (-3.1 to +1.6) | .540 |
| Complete-case categorical analysis | Medium vs high | +0.3 (-1.9 to +2.4) | .802 |
|  | Low vs high | +0.3 (-1.5 to +2.1) | .750 |
| Multiple imputation | Medium vs high | +0.3 (-1.8 to +2.4) | .757 |
|  | Low vs high | +0.2 (-1.7 to +2.2) | .818 |
| Continuous log-income analysis | P75 vs P25 (weighted P25 approximately 202 and P75 approximately 433 x10,000 JPY) | -0.5 (-0.9 to +0.0) | .075 |

*The primary analysis treated missing income as a category. Complete-case and multiple-imputation analyses used the 3-level high, medium, and low categorization. The continuous model used log(equivalized household income + 1). Multiple imputation used 20 imputations and Rubin-rule pooling.*

Table S10. Composition and refined analysis of the baseline unemployed category.

| **Panel A. Composition of the original unemployed category** | | | | | | | | | | | | | | |
| --- | --- | --- | --- | --- | --- | --- | --- | --- | --- | --- | --- | --- | --- | --- |
| **Subgroup** | **n** | | | **Weighted share, %** | **Weighted mean age, y** | | | **Weighted male, %** | | **2020 weighted prevalence, %** | | **2024 weighted prevalence, %** | | |
| Students | 227 | | | 7.0 | 20.6 | | | 40.1 | | 3.8 | | 11.7 | | |
| Retired | 563 | | | 10.5 | 70.1 | | | 70.8 | | 1.9 | | 2.6 | | |
| Homemakers | 1,874 | | | 48.4 | 56.6 | | | 3.4 | | 1.4 | | 3.2 | | |
| Other not employed | 1,468 | | | 34.0 | 60.9 | | | 61.7 | | 1.2 | | 3.7 | | |
| **Panel B. Refined repeated-wave occupational analysis** | | | | | | | | | | | | | | |
| **Baseline occupational group** | | **2020 adjusted prevalence, % (95% CI)** | | | | **2024 adjusted prevalence, % (95% CI)** | | | **Change, pp (95% CI)** | | **DID, pp (95% CI)** | | | **Adjusted P value** |
| Upper non-manual workers | | 3.8 (2.8 to 4.8) | | | | 9.6 (7.8 to 11.4) | | | +5.8 (+4.5 to +7.0) | | Reference | | |  |
| Lower non-manual workers | | 2.4 (1.3 to 3.5) | | | | 8.0 (5.5 to 10.5) | | | +5.6 (+3.5 to +7.7) | | -0.2 (-2.6 to +2.2) | | | .870 |
| Manual workers | | 2.2 (0.9 to 3.5) | | | | 11.7 (6.5 to 16.9) | | | +9.5 (+4.9 to +14.1) | | +3.7 (-1.2 to +8.7) | | | .197 |
| Self-employed | | 5.2 (2.6 to 7.7) | | | | 9.3 (6.5 to 12.1) | | | +4.1 (+2.3 to +5.9) | | -1.7 (-3.7 to +0.4) | | | .196 |
| Students | | 2.1 (0.6 to 3.6) | | | | 6.6 (2.3 to 10.8) | | | +4.5 (+0.9 to +8.0) | | -1.3 (-5.4 to +2.7) | | | .608 |
| Retired | | 3.0 (0.6 to 5.4) | | | | 4.1 (1.3 to 7.0) | | | +1.1 (-0.2 to +2.5) | | -4.7 (-6.1 to -3.2) | | | .002 |
| Homemakers | | 2.1 (1.0 to 3.1) | | | | 4.6 (2.8 to 6.4) | | | +2.6 (+1.1 to +4.0) | | -3.2 (-5.1 to -1.4) | | | .002 |
| Other not employed | | 1.4 (0.7 to 2.2) | | | | 4.3 (2.4 to 6.1) | | | +2.8 (+1.4 to +4.3) | | -2.9 (-5.0 to -0.9) | | | .012 |
| **Panel C. New adoption among 2020 never users** | | | | | | | | | | | | | | |
| **Baseline occupational group** | | | **Adjusted new-adoption risk, % (95% CI)** | | | | **Risk difference vs upper non-manual workers, pp (95% CI)** | | | | | | **Adjusted P value** | |
| Upper non-manual workers | | | 6.1 (4.6 to 7.5) | | | | Reference | | | | | |  | |
| Lower non-manual workers | | | 5.3 (3.4 to 7.2) | | | | -0.8 (-3.1 to +1.5) | | | | | | .528 | |
| Manual workers | | | 8.7 (4.4 to 12.9) | | | | +2.6 (-1.9 to +7.1) | | | | | | .357 | |
| Self-employed | | | 4.8 (2.8 to 6.8) | | | | -1.3 (-3.5 to +0.9) | | | | | | .357 | |
| Students | | | 4.6 (0.7 to 8.6) | | | | -1.4 (-5.9 to +3.0) | | | | | | .528 | |
| Retired | | | 1.7 (-0.5 to 4.0) | | | | -4.3 (-6.6 to -2.1) | | | | | | .002 | |
| Homemakers | | | 2.5 (1.1 to 3.8) | | | | -3.6 (-5.6 to -1.6) | | | | | | .002 | |
| Other not employed | | | 3.5 (1.6 to 5.4) | | | | -2.6 (-5.0 to -0.2) | | | | | | .075 | |

*All occupational categories were measured in 2020. Models were fully adjusted, weighted, and clustered by prefecture. For Panel C, analyses were restricted to participants who reported no telemedicine use in 2020. Adjusted new-adoption risks and risk differences versus upper non-manual workers were estimated from a weighted logistic regression model using predictive margins and the same covariates as the primary occupational model. No group-by-wave cell contained zero events, and both refined occupational models converged. Benjamini-Hochberg false-discovery-rate-adjusted P values were calculated across the 7 nonreference occupational contrasts within each analysis.*

Table S11. Secondary analysis of past-year telemedicine use in 2024 among participants who were never users in 2020.

| **Exposure** | **Comparison** | **2024 past-year use among 2020 never users, RD pp (95% CI)** |
| --- | --- | --- |
| Age group | 30–39 vs 18–29 years | -3.2 (-7.6 to +1.2); adjusted P=.289 |
|  | 40–49 vs 18–29 years | -3.8 (-8.1 to +0.6); adjusted P=.192 |
|  | 50–59 vs 18–29 years | -4.4 (-8.6 to -0.2); adjusted P=.130 |
|  | 60–69 vs 18–29 years | -5.8 (-9.9 to -1.7); adjusted P=.030 |
|  | 70–75 vs 18–29 years | -6.3 (-10.5 to -2.1); adjusted P=.030 |
| Educational attainment | 2-year college vs university or higher | -0.8 (-2.2 to +0.6); adjusted P=.433 |
|  | High school or lower vs university or higher | -0.2 (-1.6 to +1.2); adjusted P=.840 |
| Urbanicity | Rural vs urban | +0.4 (-0.8 to +1.6); adjusted P=.655 |
| Household income | Medium vs high | -0.2 (-1.5 to +1.1); adjusted P=.840 |
|  | Low vs high | -0.5 (-2.2 to +1.1); adjusted P=.655 |
|  | Not answered vs high | -1.8 (-3.7 to +0.0); adjusted P=.130 |
| Occupational class | Lower non-manual vs upper non-manual | -1.7 (-2.9 to -0.5); adjusted P=.030 |
|  | Manual vs upper non-manual | +2.3 (-1.5 to +6.1); adjusted P=.385 |
|  | Self-employed vs upper non-manual | -0.1 (-1.9 to +1.7); adjusted P=.905 |
|  | Unemployed vs upper non-manual | -1.3 (-2.6 to +0.0); adjusted P=.130 |

*Analyses were restricted to participants who had never used telemedicine in 2020. Past-year telemedicine use in 2024 was defined by response options 1–3. Adjusted risk differences (RDs) were estimated using predictive margins from weighted logistic regression models with the same covariate adjustment set as the corresponding primary models and standard errors clustered by prefecture. Benjamini–Hochberg false-discovery-rate-adjusted P values were calculated across the 15 nonreference contrasts.*

# Figures

Figure S1. Adjusted prevalence of telemedicine adoption in 2020 and 2024 by continuous baseline age.

*Lines show marginally standardized adjusted prevalence from a fully adjusted inverse-probability-weighted logistic model. Baseline age was modeled using a restricted cubic spline with knots at ages 25, 47, 62, and 75 years. Shaded areas and dashed boundaries indicate 95% confidence intervals. Standard errors were clustered by prefecture.*

Figure S2. Adjusted change in telemedicine adoption from 2020 to 2024 by continuous baseline age.

*The solid line shows the adjusted within-age change in predictive margins between 2020 and 2024; the shaded area and dashed boundaries indicate the 95% confidence interval. Baseline age was modeled using a restricted cubic spline with knots at ages 25, 47, 62, and 75 years. The horizontal dashed line at 0 indicates no change. The model was fully adjusted, inverse-probability weighted, and clustered by prefecture.*
